# Prediction of hepatocellular carcinoma risk in patients with chronic liver disease from dynamic modular networks

**DOI:** 10.1186/s12967-021-02791-9

**Published:** 2021-03-23

**Authors:** Yinying Chen, Wei Yang, Qilong Chen, Qiong Liu, Jun Liu, Yingying Zhang, Bing Li, Dongfeng Li, Jingyi Nan, Xiaodong Li, Huikun Wu, Xinghua Xiang, Yehui Peng, Jie Wang, Shibing Su, Zhong Wang

**Affiliations:** 1 Guang’anmen Hospital, China Academy of Chinese Medical Sciences, No. 5 Beixian Ge, Xicheng District, Beijing, 100053 China; 2 Institute of Basic Research in Clinical Medicine, China Academy of Chinese Medical Sciences, Dongzhimen, Beijing, 100700 China; 3 Postdoctoral Research Station, China Academy of Chinese Medical Sciences, Beijing, China; 4 Research Center for Traditional Chinese Medicine Complexity System, Institute of Interdisciplinary Integrative Medicine Research, Shanghai University of Traditional Chinese Medicine, 1200 Cailun Road, Pudong, Shanghai, 201203 China; 5 School of Mathematical Sciences, Peking University, Beijing, China; 6 Shandong Danhong Pharmaceutical Co. Ltd., Heze, China; 7 Hubei Provincial Hospital of Traditional Chinese Medicine, Wuhan, China; 8 School of Mathematics and Computational Science, Hunan University of Science and Technology, Xiangtan, China

**Keywords:** Chronic liver disease, Hepatocellular carcinoma (HCC), Chronic hepatitis B (CHB), Cirrhosis, Dynamic modular networks, Sequential allosteric modules, HCC risk

## Abstract

**Background:**

Discovering potential predictive risks in the super precarcinomatous phase of hepatocellular carcinoma (HCC) without any clinical manifestations is impossible under normal paradigm but critical to control this complex disease.

**Methods:**

In this study, we utilized a proposed sequential allosteric modules (*AMs*)-based approach and quantitatively calculated the topological structural variations of these *AM*s.

**Results:**

We found the total of 13 oncogenic allosteric modules (*OAMs*) among chronic hepatitis B (CHB), cirrhosis and HCC network used SimiNEF. We obtained the 11 highly correlated gene pairs involving 15 genes (r > 0.8, P < 0.001) from the 12 *OAMs* (the out-of-bag (OOB) classification error rate < 0.5) partial consistent with those in independent clinical microarray data, then a three-gene set (cyp1a2-cyp2c19-il6) was optimized to distinguish HCC from non-tumor liver tissues using random forests with an average area under the curve (AUC) of 0.973. Furthermore, we found significant inhibitory effect on the tumor growth of Bel-7402, Hep 3B and Huh7 cell lines in zebrafish treated with the compounds affected those three genes.

**Conclusions:**

These findings indicated that the sequential *AMs*-based approach could detect HCC risk in the patients with chronic liver disease and might be applied to any time-dependent risk of cancer.

**Supplementary Information:**

The online version contains supplementary material available at 10.1186/s12967-021-02791-9.

## Background

Hepatocellular carcinoma (HCC) is the most common primary liver cancer with poor prognosis. Many factors are considered to contribute to hepatitis B virus (HBV)-associated HCC, including the aberrant expression of microRNAs [1], aberrant DNA methylation [2], mutated genes [3], alterations in multiple signaling pathways and host gene expression [4–6]. Some serum or tissue biomarkers for the diagnosis of HCC have been successfully identified [7]. However, previous research has focused on identifying risk of preclinical HCC for screening the early presence of premalignant lesions and tumors [8]. Despite progress in diagnostics and treatment of HCC, its prognosis remains poor [9, 10].

Evidence suggests that there is usually a critical transition point during disease progression, resulting in the critical transition from a normal state to a disease state. Therefore, it is very important to detect the early warning signals of the predisease state to prevent sudden deterioration [11]. Thus, can we identify predictive risk for HCC at an earlier stage?

From the perspective of Modular Pharmacology (MP), the treatment of complex diseases requires a modular design to affect multiple targets [12]. The exploration of modular structure has been a key factor in understanding the complexity of disease networks [13]. A disease module represents a cellular function whose disruption results in a particular disease phenotype [13]. In our previous study, we proposed the concept of allosteric modules (*AMs*), which refers to multipotent functional changes in modular architecture [14]. Allostery controls pathway divergence and unification and encodes specific effects on cellular pathways [15, 16]. The fundamental importance of allostery is the exertion of functional effects on signaling pathways and the entire cellular network [16, 17]. The *AMs* may provide valuable structural information about disease and pharmacological networks beyond pathway analysis.

In this study, by integrating the multi-source data (including *AM*s, clinical microarray data and The Cancer Genome Atlas [TCGA] dataset), we constructed risk prediction models and proposed the sequential *AMs* -based approach for predicting the risk of HCC in patients with chronic liver disease.

## Methods

### Constructing disease-associated networks for each pathological stage

A list of disease-associated genes was obtained from the Online Mendelian Inheritance in Man (OMIM) database (http://www.ncbi.nlm.nih.gov/omim), including 220 hepatitis B-related genes, 152 liver cirrhosis-related genes, and 213 HCC-related genes. We used disease-associated genes from OMIM to construct 3 global disease-associated networks using the Agilent literature search plugin in Cytoscape.

### Identifying and optimizing functional modules in different groups

In each disease-associated network, functional modules were identified using the Molecular Complex Detection (MCODE) algorithm [18]. For MCODE, we tried all possible combinations of the following parameters: Include Loops: false; Degree Cutoff: 3; Node Score Cutoff: 0.0, 0.2, 0.3; Haircut: true or false; Fluff: true or false; K-Core: 2; and Max Depth from Seed: 100, 5, 4, 3. A total of 48 parameter combinations were calculated. After the functional modules were identified, they were optimized according to the minimum entropy criterion, and the analysis of calculating minimal network entropy was carried out as described previously [14].

### Calculating the similarities of the *AM*s

The similarities of the nodes and edges of the modules were calculated with our proposed method of SimiNEF [14]. Briefly, we used similarity *S*_*ne*_ to quantify the relative overlaps between *AM*s *m*_*i*_ and *m*_*j*_, including the overlaps of nodes and edges together. The similarities of nodes *S*_*n*_ (*m*_*i*_, *m*_*j*_) and edges *S*_*e*_ (*m*_*i*_, *m*_*j*_) are defined in Eqs. 1 and 2, respectively.

1 $${S}_{n} ({m}_{i},{m}_{j})=\frac{\left|N({m}_{i})\cap N({m}_{j})\right|}{\left|N({m}_{i})\cup N({m}_{j})\right|}$$

2 $${S}_{e} ({m}_{i},{m}_{j})=\frac{\left|E({m}_{i})\cap E({m}_{j})\right|}{\left|E({m}_{i})\cup E({m}_{j})\right|}$$

### Enrichment analysis of KEGG pathways

The enrichment analysis of KEGG pathways in the modules was performed using a hypergeometric test, as implemented on the KOBAS 2.0 web server (http://kobas.cbi.pku.edu.cn/) [19].

### Clinical microarray data

#### Clinical samples and RNA extraction

Morning fasting venous blood samples from a total of 36 patients were obtained from Shuguang Hospital and Longhua Hospital in Shanghai, China, including 3 healthy people, 10 chronic hepatitis B (CHB) patients, 13 HBV-related cirrhosis (cirrhosis) patients and 10 HCC patients. The research protocol was approved by the respective Institutional Review Boards. The study was approved by the Official Ethics Committee of the Shanghai University of Traditional Chinese Medicine, and written informed consent was obtained from all study participants. Chronic hepatitis B, HBV-related cirrhosis and HCC were diagnosed according to the “Chronic hepatitis B prevention and treatment guidelines” [20], “Standard of clinic diagnosis, syndrome differentiation and assessing curative effect on hepatocirrhosis” [21], and “clinical diagnosis and staging criteria for primary hepatocellular carcinoma” established by the Chinese Society of Liver Cancer in 2001 [22], respectively.

The microarray methods followed those described in previous studies [23–25]. The leukocytes were isolated from the blood samples by Ficoll optimized density gradient separation and stored at − 80 ℃ [26]. Total RNA was extracted using a “two-step” protocol as described previously. Total RNA from leukocytes from whole blood was extracted using TRIzol reagent according to the manufacturer’s instructions (Invitrogen, Carlsbad, CA, USA) and stored at − 80 ℃. The quantity and quality of RNA were assessed using a NanoDrop ND-1000 spectrophotometer (NanoDrop Technology, Rockland, DE).

#### Microarray data analysis

Briefly, cDNA was synthesized by the Invitrogen First-Strand cDNA Synthesis Kit (Invitrogen, Carlsbad, CA, USA), and RNA polymerase was added to degrade RNA. The biotinylated cDNAs were labeled and hybridized to a NimbleGen Homo sapiens 12 × 135K gene expression array (Roche, Cat No. A6484-00-01). After hybridization and washing, the processed slides were scanned with the Axon GenePix 4000B microarray scanner (Molecular Devices, Sunnyvale, CA). Raw data were extracted as pair files by NimbleScan software (version 2.5), and the data were considered robustly expressed if the signal/noise ratio (SNR) > 2. NimbleScan software’s implementation of the robust multiarray analysis (RMA) algorithm offers the quantile normalization and background correction of data. The gene summary files were imported into Agilent GeneSpring Software (version 11.0, Agilent, USA) for further analysis. Both the P-value significance of *t*-test and the fold-change directionality (up- or downregulation) were taken into consideration for identifying differentially expressed genes between the two groups. Genes with a P-value < 0.05 and a fold-change > 1.5 or < − 1.5 were considered differentially expressed.

### Construction of random forests models and rule extraction for predicting HCC

First, by combining genes in the *OAMs* with microarray data, we used the random forests algorithm to model and predict chronic hepatitis B, cirrhosis and HCC. The random forests algorithm was run independently on each of the *OAMs.* Then, the out-of-bag (OOB) error rates of the random forests models were computed. The variables of the model leading to the smallest OOB error were selected. The random forests algorithm has been extensively used to rank variable importance, i.e., genes. In this study, the Gini index was used as a measurement of predictive performance and a gene with a large mean decrease in Gini index (MDG) value is more important than a gene with a small MDG. The importance of the genes in discriminating HCC from non-tumor samples was evaluated by the MDG values.

Second, we further explored the predictive performance of the important genes for HCC by using The Cancer Genome Atlas (TCGA) database for the liver hepatocellular carcinoma (LIHC) project (https://portal.gdc.cancer.gov/projects/TCGA-LIHC). Human HCC mRNA-seq data were downloaded, containing 374 HCC tumor tissues and 50 adjacent non-tumor liver tissues. Receiver operating characteristic (ROC) curves and the associated area under the curve (AUC) values of the important genes were generated to evaluate their capacity to distinguish non-tumor tissues from HCC samples. An AUC value close to 1 indicates that the test classifies the samples as tumor or non-tumor correctly, while an AUC of 0.5 indicates no predictive power. In addition, The G-mean was used to consider the classification performance of HCC and non-tumor samples at the same time; The F-value, Sensitivity and Precision were used to consider the classification power of HCC; The Specificity is used to consider the classification power of normal; Accuracy is used to indicate the performance of all categories correctly. In particular, the intergroup differences of classification evaluation indexes between two-gene and three-gene combinations were evaluated using the normal *t*-test or nonparametric Mann–Whitney *U* test.

The data analysis in this paper is implemented by R software. We used RandomForest function in the randomForest package and these functions (RF2List, extractRules, unique, getrulemors, pruneRule, selectRuleRRF, buildLearner, applyLearner, presentRules) in the inTrees package. All parameters of functions were set by default.

Next, we used rule extraction to establish the conditions of the three genes to correctly predict HCC. We applied the inTrees (interpretable trees) framework to extract interpretable information from tree ensembles [27]. A total of 1780 rule conditions extracted from the first 100 trees with a maximum length of 6 were selected from random forests by the condition extraction method in the inTrees package. Leave-one-out pruning was applied to each variable-value pair sequentially. In the rule selection process, we applied the complexity-guided regularized random forest algorithm to the rule set (with each rule being pruned).

### Experimental verification

We screened related compounds that affected the three genes (cyp1a2-cyp2c19-il6). Then, the drug combination containing the corresponding compounds was used to treat three different human HCC cell lines (Bel-7402, Hep 3B and Huh7). Bel-7402, Hep 3B and Huh7 cells were labeled with green fluorescent dye and transplanted into the yolk sac of wild-type AB strain zebrafish 2 days after fertilization (2 dpf) by microinjection. About 200 cells were transplanted into each fish to establish a zebrafish human HCC transplantation model. Zebrafishes injected with human HCC cells were cultured at 35 ℃ to 3 dpf. At 3 dpf, zebrafishes with good consistency of transplanted tumor were selected under the microscope and randomly distributed into 6-well plates with 30 fishes per well. In experimental groups, Jiangan (JG) granules were given with water-soluble concentrations of 27.8, 83.3 and 250 µg/mL, respectively. The positive control group was treated by cisplatin (15 µg/mL). And the vehicle group was set. Ten zebrafishes for each group were randomly selected to collect the fluorescence intensity of transplanted tumor. The statistical analysis results of fluorescence intensity were used to evaluate the growth inhibition effect of JG granules on human HCC transplanted tumor in the zebrafish model.

## Results

### Constructing disease-associated networks for each pathological stage

**Fig. 1:**
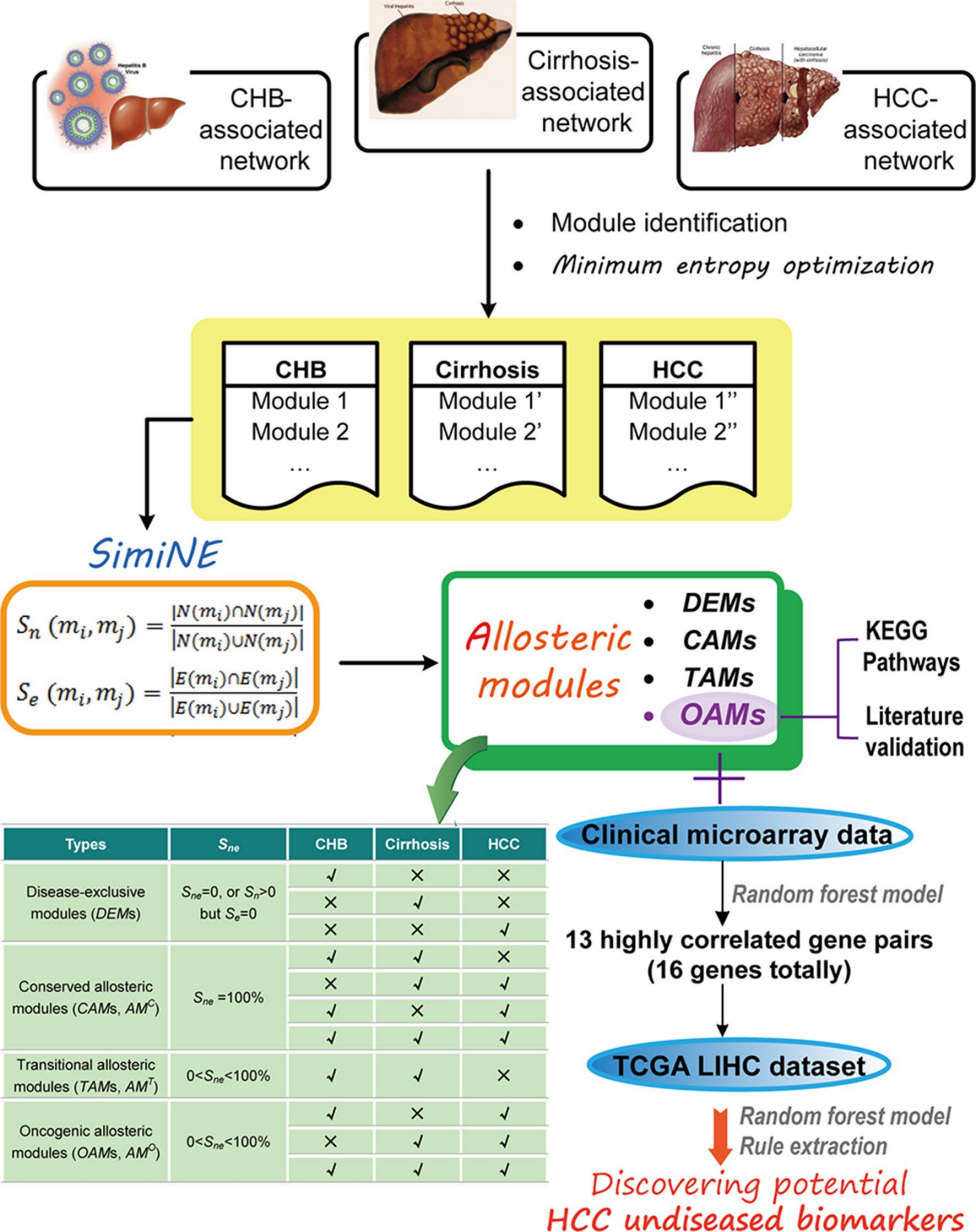
Flow diagram. CHB-, cirrhosis-, and HCC-associated networks were constructed using disease-associated genes downloaded from OMIM. Functional modules were identified using the MCODE algorithm. Then, the results of module identification were optimized based on the minimum entropy criterion. The enrichment analysis of KEGG pathways was performed with DAVID 6.7 software. The similarity between modules was calculated using SimiNEF. Four *AM*s (*DEM*s, *CAM*s, *TAM*s, and *OAM*s) were identified. The relationships between *OAM* genes and HCC were validated by published literature. *AM*s allosteric modules, *DEM*s disease-exclusive modules, *CAM*s conserved allosteric modules, *TAM*s transitional allosteric modules, and *OAM*s oncogenic allosteric modules. ‘√’ or ‘×’ represents its appearance ‘yes’ or ‘no’ in the group, respectively. For example, the module is identified as ‘conserved’ when it is found both in CHB and cirrhosis, cirrhosis and HCC, CHB and HCC, or among the three groups (‘√’), and *S*_*ne*_ = 100%

A schematic diagram of the entire analysis framework is shown in Fig. 1. CHB-, cirrhosis-, and HCC-associated networks were constructed, involving 1104, 487, and 1079 nodes, respectively (Additional file 1: Table S1). The cirrhosis-associated network had the minimum number of nodes, and there was only a small difference in network size between the other two networks (Additional file 1: Table S1). Therefore, an analysis of the entire networks might not be sufficient to reveal the pathophysiological changes from chronic hepatitis to HCC.

### Identifying and optimizing functional modules

The results identified by MCODE are shown in Additional file 1: Table S2. Considering the influence of different parameters on the clustering results, we tested 48 parameter settings. After the optimization of minimum entropy, 53, 21, and 60 modules (nodes ≥ 4) were identified from CHB-, cirrhosis-, and HCC-associated networks, respectively (Additional file 1: Table S1). The average sizes of these modules ranged from 4.609 to 6.447, and the entropy values were similar between the CHB- and HCC-associated networks after module optimization (Additional file 1: Table S1).

### Difference gradient among the *AM*s of the three pathological stages

**Fig. 2:**
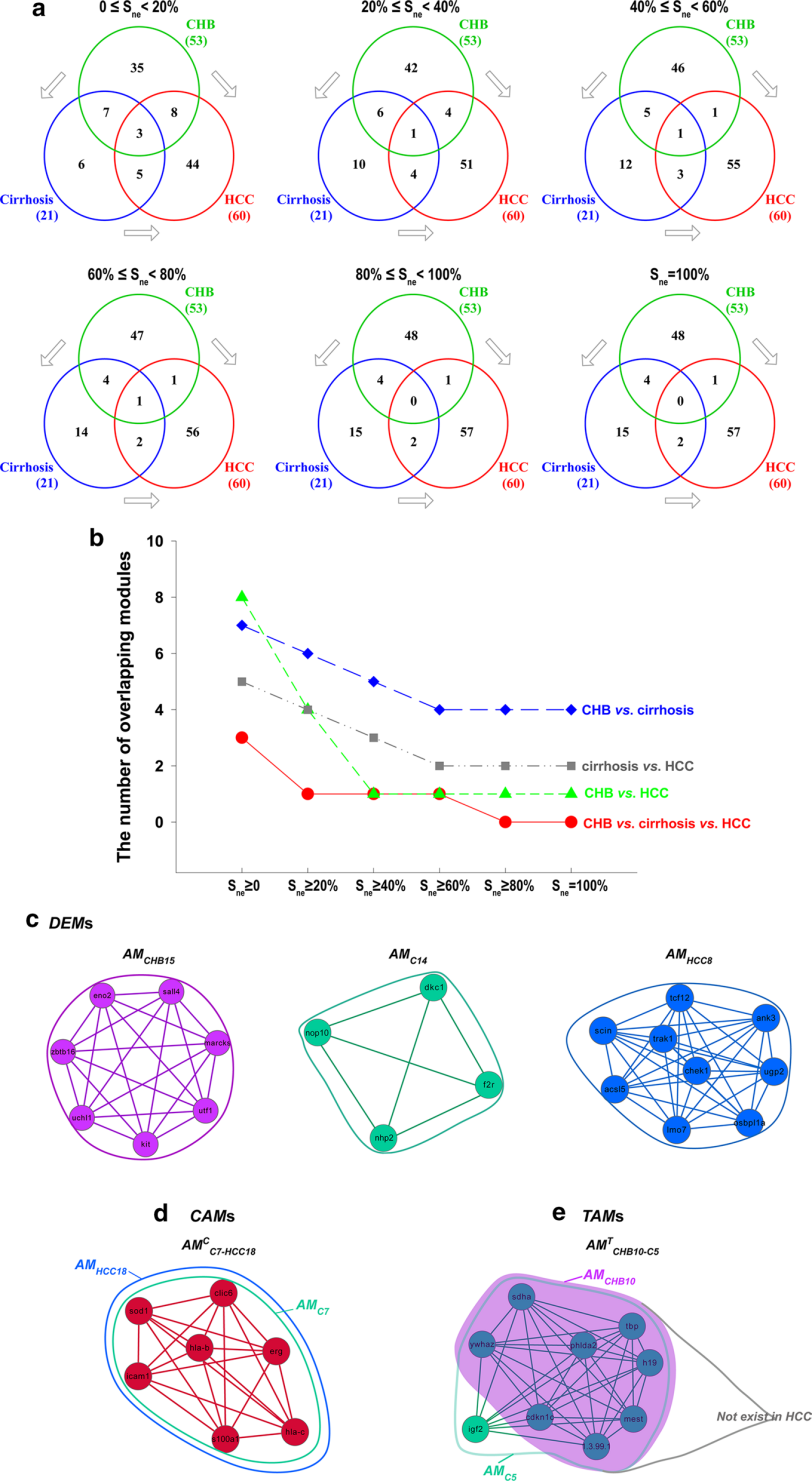
Different levels of similarities between the modules in the CHB, cirrhosis and HCC groups. **a** Six levels of similarities reflecting the degree of overlap between modules in the CHB, cirrhosis and HCC groups, including *S*_*ne*_ > 0, > 20%, > 40%, > 60%, > 80%, and = 100%. Each Venn diagram represents a level of similarity. The green circle denotes the number of modules in the CHB group. The blue circle denotes the number of modules in the cirrhosis group. The red circle denotes the number of modules in the HCC group. Gray arrows indicate the progression of the disease. **b** The changing trends of the number of overlapping modules between the CHB and cirrhosis groups (blue line), the cirrhosis and HCC groups (gray line), the CHB and HCC groups (green line), and among the three groups (red line). **c** Examples of *DEM*s. *AM*_*CHB15*_, *AM*_*C14*_, and *AM*_*HCC8*_ were *DEM*s in the CHB, cirrhosis and HCC groups, respectively. **d** Examples of *CAM*s. The area in the blue solid line represents *AM*_*HCC18*_. The green solid line area represents *AM*_*C7*_. **e** Examples of *TAM*s. The green solid line area represents *AM*_*C5*_. The purple area indicates *AM*_*CHB10*_

We used similarity *S*_*ne*_ > 0, > 20%, > 40%, > 60%, > 80%, and = 100% to define the overlap between *AM*s. Hence, we obtained different degrees of differences between the *AM*s (Fig. 2a). For example, it should be noted that *S*_*ne*_ > 20% means *S*_*n*_ > 20% and *S*_*e*_ > 20% simultaneously. *When S*_*ne*_ = 0 or *S*_*n*_ > 0 but *S*_*e*_ = 0, these modules are referred to as disease-exclusive modules (*DEM*s); that is, the module did not overlap with any other module from other groups (Figs. 1, 2c). There were 35, 6, and 44 *DEM*s in the CHB, cirrhosis, and HCC groups, respectively (Fig. 2a). The results showed that from *S*_*ne*_ ≥ 0 to *S*_*ne*_ = 100% in 20% increments, the number of overlapping modules among the CHB, cirrhosis and HCC groups was 3, 1, 1, 1, 0, and 0; the number of overlapping modules between the CHB and cirrhosis groups was 7, 6, 5, 4, 4, and 4; the number of overlapping modules between the CHB and HCC groups was 8, 4, 1, 1, 1, and 1; and the number of overlapping modules between the cirrhosis and HCC groups was 5, 4, 3, 2, 2, and 2, respectively, showing a gradual decreasing trend. In other words, with the increments of *S*_*ne*_, the degree of difference among *AM*s increased gradually (Fig. 2a, b). When *S*_*ne*_ > 80% and *S*_*ne*_ = 100%, there were no overlapping modules among the three groups (Fig. 2a, b).

### Distribution of the different *AMs* of the three pathological stages

**Fig. 3:**
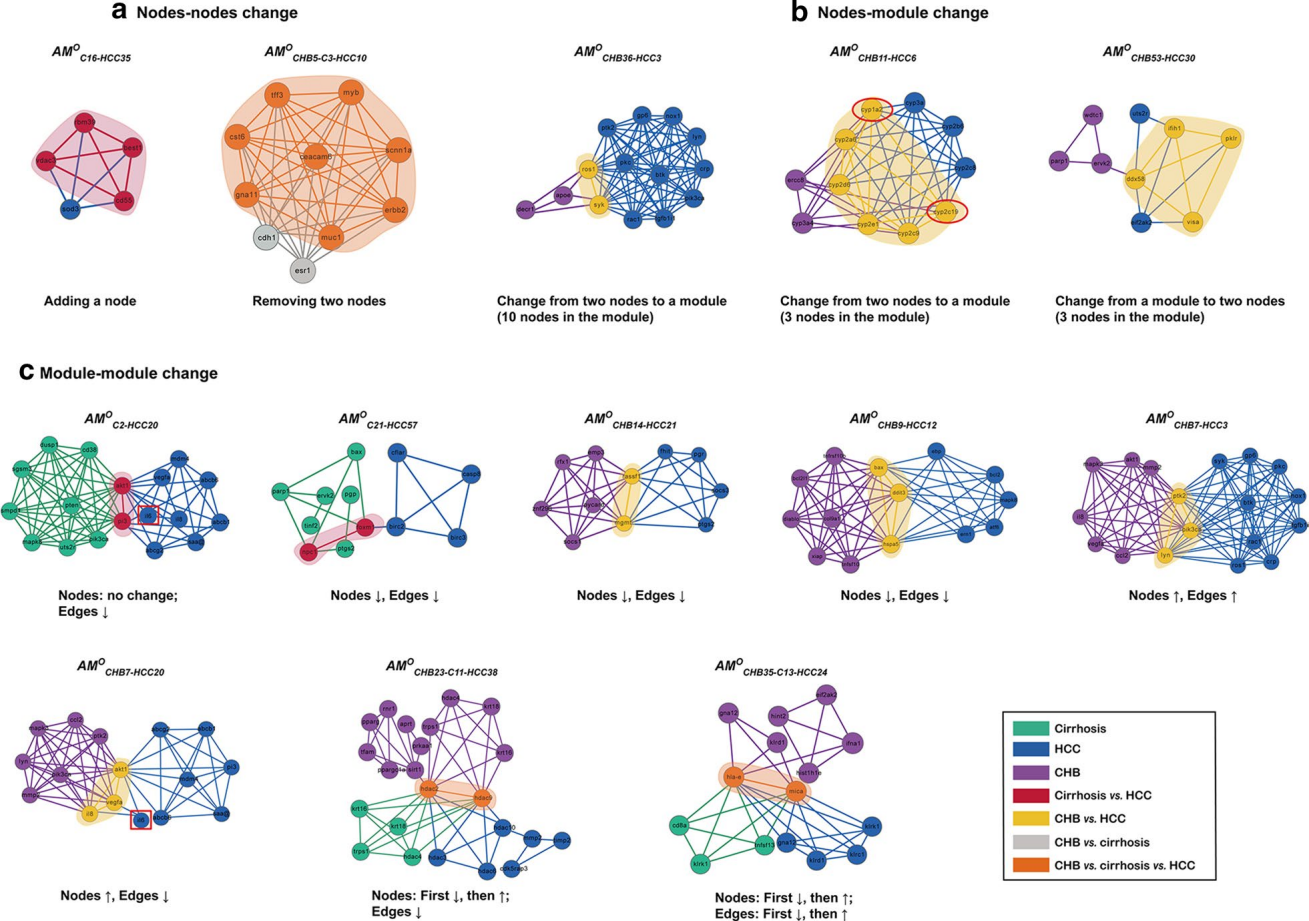
Topological changes in the 13 potential *OAM*s. The modules are color coded; green nodes and edges denote cirrhosis modules, blue nodes and edges denote HCC modules, and purple nodes and edges denote CHB modules. The overlapping nodes and edges between modules are highlighted in red (cirrhosis vs. HCC), yellow (CHB vs. HCC), gray (CHB vs. cirrhosis) and orange (CHB vs. cirrhosis vs. HCC), respectively. **a** Node–node change; **b** Node-module change; **c** Module-module change. The changing characteristics of the module structures in each *OAM* are listed below the modules. “↑” denotes that the number of nodes or edges increases. “↓” denotes that the number of nodes or edges decreases

Based on the changes in nodes and edges, the comparison of these modules in different disease stages resulted in three types of *AM*s (Fig. 1). (1) Conserved allosteric modules (*CAM*s, *AM*^*C*^). If the modular overlap between the CHB and cirrhosis groups, the cirrhosis and HCC groups, the CHB and HCC groups, or among the three groups reached 100% (*S*_*ne*_ = 100%), these modules were referred to as *CAM*s (Figs. 1, 2d). A total of 7 *CAM*s were identified, including *AM*^*C*^_*CHB1-C1*_, *AM*^*C*^_*CHAB5-C3*_, *AM*^*C*^_*CHB8-C4*_, *AM*^*C*^_*CHB16-C6*_, *AM*^*C*^_*CHB20-HCC25*_, *AM*^*C*^_*C7-HCC18*_, and *AM*^*C*^_*C19-HCC49*_. (2) Transitional allosteric modules (*TAM*s, *AM*^*T*^). Some partially overlapping modules (0 < *S*_*ne*_ < 100%) were identified only between the CHB and cirrhosis groups and could not be found in HCC; these modules were referred to as *TAM*s (Figs. 1, 2e). Four *TAM*s were identified, including *AM*^*T*^_*CHB10-C5*_, *AM*^*T*^_*CHB6-C2*_, *AM*^*T*^_*CHB53-C21*_, and *AM*^*T*^_*CHB7-C2*_. (3) Oncogenic allosteric modules (*OAM*s, *AM*^*O*^). Many modules partially overlapped (0 < *S*_*ne*_ < 100%) between the CHB and HCC groups, the cirrhosis and HCC groups, or among the three groups, and these modules were referred to as potential *OAM*s (Figs. 1, 3). A total of 13 *OAMs* were found, including 3 *OAMs* (*AM*^*O*^_*C2-HCC20*_, *AM*^*O*^_*C21-HCC57*_, and *AM*^*O*^_*C16-HCC35*_) between the cirrhosis and HCC groups, 7 *OAMs* (*AM*^*O*^_*CHB53-HCC30*_, *AM*^*O*^_*CHB11-HCC6*_, *AM*^*O*^_*CHB7-HCC20*_, *AM*^*O*^_*CHB9-HCC12*_, *AM*^*O*^_*CHB7-HCC3*_, *AM*^*O*^_*CHB14-HCC21*_, and *AM*^*O*^_*CHB36-HCC3*_) between the CHB and HCC groups, and 3 *OAMs* (*AM*^*O*^_*CHB5-C3-HCC10*_, *AM*^*O*^_*CHB23-C11-HCC38*_, and *AM*^*O*^_*CHB35-C13-HCC24*_) among the three groups (Fig. 3).

### Topological variations in potential *OAM*s

Next, we focused on the topological variations of the 13 potential *OAM*s. As shown in Fig. 3, a partially overlapping structure existed in each *OAM* that served as a bridge between modules, generally including the following four types. (1) One-edge overlap, wherein an edge between two nodes overlapped between two modules. Six *OAM*s (*AM*^*O*^_*CHB36-HCC3*_, *AM*^*O*^_*C2-HCC20*_, *AM*^*O*^_*C21-HCC57*_, *AM*^*O*^_*CHB14-HCC21*_, *AM*^*O*^_*CHB23-C11-HCC38*_, and *AM*^*O*^_*CHB35-C13-HCC24*_) were included in this category (Fig. 3b, c). (2) Triangular overlap, wherein there were three overlapping nodes and edges between modules. Three *OAM*s (*AM*^*O*^_*CHB9-HCC12*_, *AM*^*O*^_*CHB7-HCC3*_, and *AM*^*O*^_*CHB7-HCC20*_) had overlapping structures (Fig. 3c). (3) Multiedge overlap, wherein there were more than three overlapping nodes and edges between modules. Two *OAM*s (*AM*^*O*^_*CHB11-HCC6*_ and *AM*^*O*^_*CHB53-HCC30*_) were included in this category (Fig. 3b). (4) Fully contained overlap, wherein one module was fully contained within the other. Two *OAM*s (*AM*^*O*^_*C16-HCC35*_ and *AM*^*O*^_*CHB5-C3-HCC10*_) had overlapping structures (Fig. 3a). One-edge overlap was the most common type, and it could be found in *OAM*s from the three paths above. Triangular overlap and multiedge overlap only existed in *OAM*s between the CHB and HCC groups (Fig. 3).

In addition, the topological changes in the nonoverlapping parts of each *OAM* also involved three situations as follows. (1) Node–node changes, wherein the modular changes included adding or removing nodes (the number of changing nodes < 3). Two *OAMs* were related to the change in nodes (Fig. 3a). (2) Node-module changes. These changes included changes from nodes (the number of changing nodes < 3) to a module (the number of changing nodes ≥ 3) or from a module to nodes. Three *OAMs* showed changes between nodes and modules (Fig. 3b). (3) Module-module changes. Eight *OAMs* were involved in the changes from module to module, indicating that the total number of nodes and edges in modules increased or decreased. Module-module changes appeared in all three carcinogenic paths (Fig. 3c).

### KEGG pathway analysis of 13 *OAM*s

**Fig. 4:**
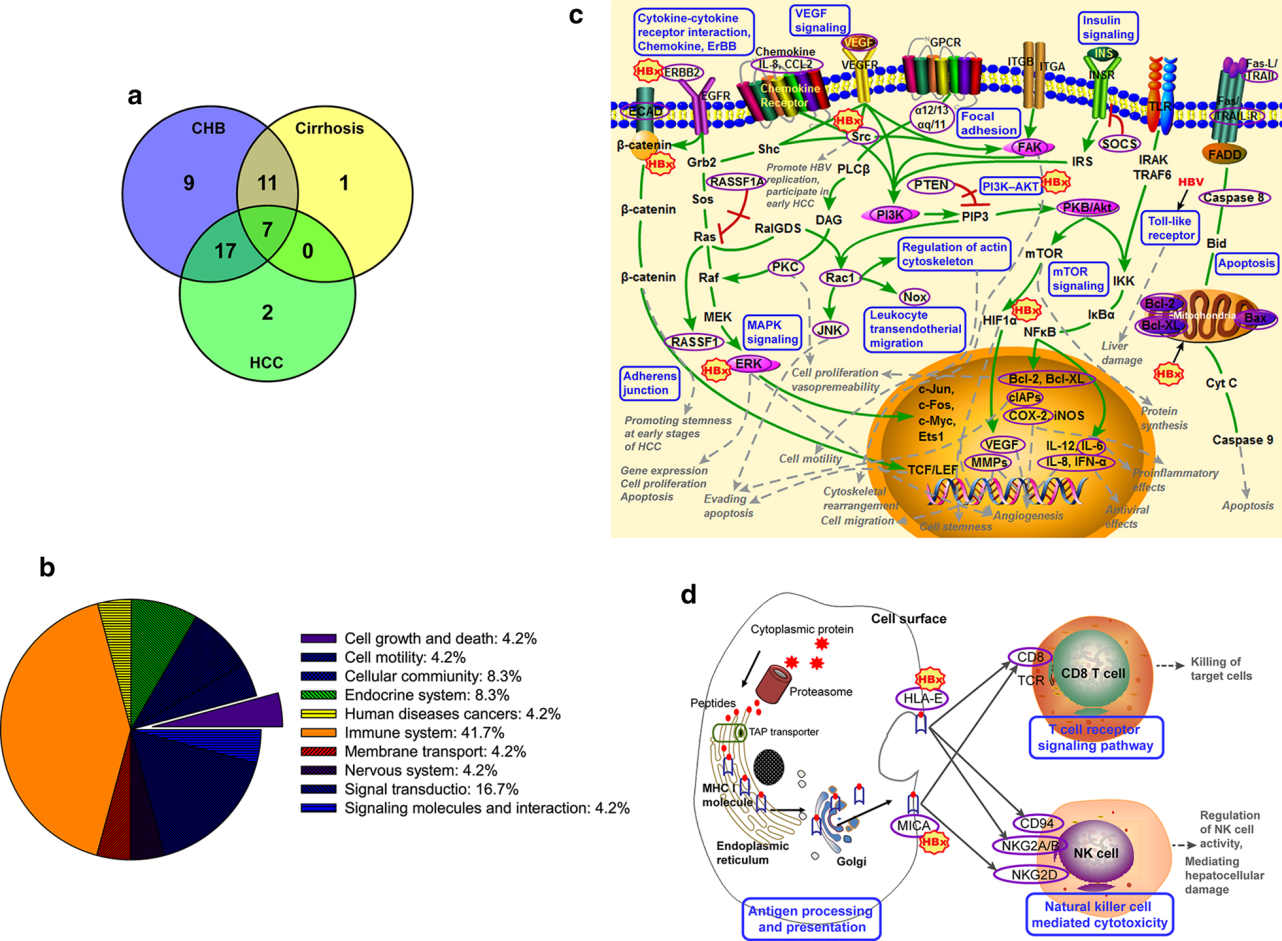
KEGG pathway analysis of the 13 *OAMs*. **a** The number of overlapping pathways among CHB, cirrhosis and HCC. **b** The 24 altered pathways were divided into 10 categories. **c**, **d** Altered signaling pathways in the progression of HBV-associated HCC. Altered pathways are denoted as blue rectangles. HBV-encoded proteins (hepatitis B x, HBx) are marked as red polygons, which stimulate/activate/influence genes adjacent to the HBx proteins. The genes in the *OAM*s are represented as purple ellipses. ECAD (CDH1), E-cadherin; ERBB2, erb-b2 receptor tyrosine kinase 2; IL-8 (IL8), interleukin 8; CCL-2 (CCL2), C–C motif chemokine 2; TRAIL (TNFSF10), tumor necrosis factor ligand superfamily member 10; TRAIL-R (TNFRSF10, TRAILR), tumor necrosis factor receptor superfamily member 10; Src (SRC), tyrosine-protein kinase Src; α12/13 (GNA12), guanine nucleotide-binding protein subunit alpha-12; αq/11 (GNA11), guanine nucleotide-binding protein subunit alpha-11; SOCS (SOCS3), suppressor of cytokine signaling 3; PTEN, phosphatase and tensin homolog; FAK (PTK2), focal adhesion kinase 1; PI3K (PIK3CA), phosphatidylinositol-4,5-bisphosphate 3-kinase catalytic subunit alpha; PKB/Akt (AKT1), v-akt murine thymoma viral oncogene homolog 1; PKC, classical protein kinase C; Rac1 (RAC1, Rac-1), Ras-related C3 botulinum toxin substrate 1; Nox, NADPH oxidase; JNK (MAPK8), c-Jun N-terminal kinase, mitogen-activated protein kinase 8; RASSF1 (RASSF1A) Ras association (RalGDS/AF-6) domain family member 1; ERK (MAPK1_3), mitogen-activated protein kinase 1/3; Bcl-2 (BCL2), B-cell CLL/lymphoma 2; Bcl-XL (Bcl2l1), BCL2-like 1; cIAPs (BIRC3), baculoviral IAP repeat containing 3; COX-2 (PTGS2, COX2), prostaglandin-endoperoxide synthase 2; VEGF (VEGFA), vascular endothelial growth factor A; IL-6 (IL6), interleukin 6; MMPs (MMP2), matrix metalloproteinase-2; IFN-α (IFNA), interferon alpha; HLA-E (MHC), major histocompatibility complex, class I, E; MICA, MHC class I polypeptide-related sequence A; CD8 (CD8A), CD8a molecule; CD94 (KLRD1), killer cell lectin-like receptor subfamily D member 1; NKG2A/B (KLRC1), killer cell lectin-like receptor subfamily C, member 1; NKG2D (KLRK1), killer cell lectin-like receptor subfamily K member 1

In the 13 *OAMs*, the number of overlapping pathways between any two pathological stages (CHB, cirrhosis and HCC) was 18, 24, and 7, respectively. A total of 7 overlapping pathways were identified among the three pathological stages (Fig. 4a, Additional file 1: Table S3). KEGG pathways were restricted to those involved in biological processes. Consequently, disease pathways were discarded (except KEGG pathways related to liver disease).

After removing other disease pathways and overlapping pathways, the remaining nonoverlapping pathways were referred to as altered pathways. A total of 24 altered pathways were found during CHB-HCC progression, which could be largely divided into 10 categories, including cell growth and death (4.2%), cell motility (4.2%), cellular community (8.3%), endocrine system (8.3%), human diseases cancers (4.2%), immune system (41.7%), membrane transport (4.2%), nervous system (4.2%), signal transduction (16.7%), and signaling molecules and interaction (4.2%) (Additional file 1: Table S4, Fig. 4b). The neurotrophin signaling pathway appeared in four *OAM*s and had the highest frequency (Additional file 1: Table S4). The remaining pathways were all HCC-related pathways, except for six altered pathways that have not been previously reported to be associated with HCC (Additional file 1: Table S4).

### Reanalysis of the genes in the 13 *OAMs* with clinical microarray data

#### The consistency between the groups with differentially expressed genes and the groups represented by *OAMs*

The microarray expression data (comprising 19,471 genes) of 36 clinical samples were used. The number of overlapping genes between the CHB-, cirrhosis-, and HCC-associated networks (see section 1 of the results) and the microarray data was 989, 423, and 939 genes (accounting for 89.6%, 86.9%, and 87% of the network genes), respectively. In the microarray data, the numbers of genes significantly altered in the CHB, cirrhosis and HCC groups were 6251, 937, and 2175, respectively, compared with the normal group. The number of overlapping genes between CHB-, cirrhosis-, and HCC-associated networks and significantly altered genes in the microarray data was 279, 23, and 124 genes, respectively.

**Fig. 5:**
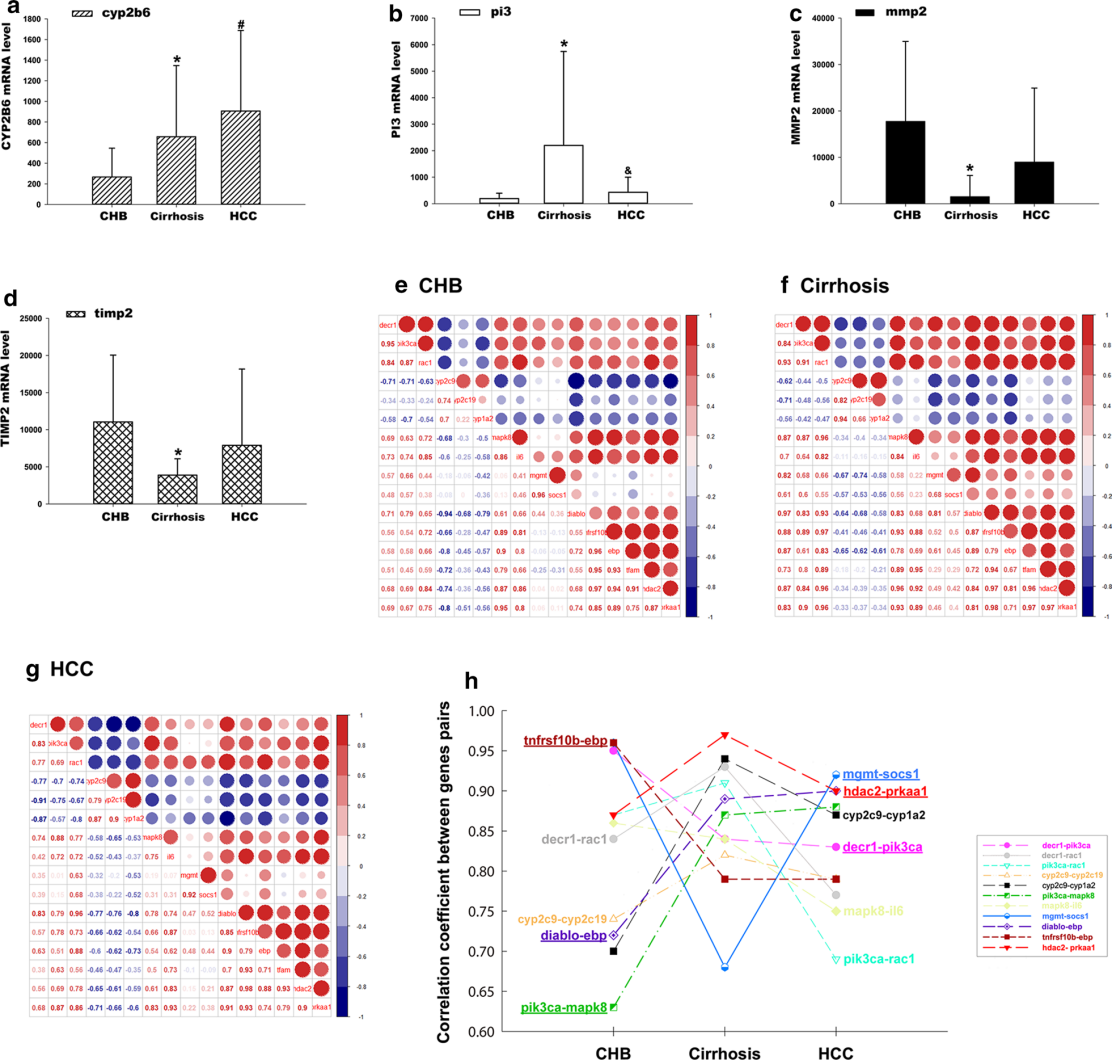
Reanalysis of the genes in the 13 *OAMs* combined with clinical microarray data. **a**–**d** The mRNA levels of *CYP2B6, PI3, MMP2* and *TIMP2* among different groups. **#** denotes statistical significance (*P* < 0.05) between the CHB and HCC groups; & denotes statistical significance (*P* < 0.05) between the cirrhosis and HCC groups; and * denotes statistical significance (*P* < 0.05) between the CHB and cirrhosis groups. **e**–**g** The correlation coefficient between the 11 pairs of genes in CHB, cirrhosis, and HCC. All gene pairs were highly correlated in the three disease states (r > 0.63). In the matrix, the red circles indicate a positive correlation, while the blue circles indicate a negative correlation. The larger a circle is, the stronger the correlation. **h** The changing trend of the correlation coefficient between the 11 pairs of genes in the three pathologic stages (CHB, cirrhosis, and HCC). The underlined gene pairs indicate that the changing trends in the correlation of 6 gene pairs in the three disease states were consistent with the disease states indicated by the *OAMs* that the gene pairs belong to

The further analysis showed that a total of 121 genes were included in the 13 *OAM*s; according to the expression levels of the 121 genes in the microarray data, a total of 7 differentially expressed genes were identified between any two groups, including *cyp2b6* (CHB vs. HCC groups), *pi3* (cirrhosis vs. HCC groups), and *mmp2, pi3, ptk2, timp2, tnfrsf10b* (CHB vs. cirrhosis groups). Furthermore, we identified 4 differentially expressed genes (*cyp2b6, pi3, mmp2,* and *timp2*) (Fig. 5a–d) that satisfied the following condition: the groups in which the differentially expressed gene existed were consistent with the groups represented by the *OAM* from which the gene was derived.

#### Selecting the top 5 important genes in *OAMs* and identifying highly correlated gene pairs

We conducted correlation analysis of the 121 genes contained in the 13 *OAMs*. First, the Pearson correlation coefficients between these genes were calculated from 36 clinical samples. Through statistical tests, we screened 273 pairs of genes that were highly correlated (r > 0.8, P-value < 0.001), of which 39 highly correlated gene pairs appeared in 10 of the 13 *OAMs* (Additional file 1: Table S5).

Then, according to the 13 *OAMs*, we constructed 13 random forests models for three disease groups and estimated the out-of-bag (OOB) classification error rate respectively. A total of 12 out of the 13 *OAMs* with an OOB classification error rate < 0.5 in predicting certain disease states are summarized in column 2–5 of Additional file 1: Table S5. Among the 12 *OAMs*, 11 *OAMs* were used to predict cirrhosis with the OOB classification error rate ≤ 0.46, 4 *OAMs* were used to predict CHB with the OOB classification error rate ≤ 0.4, and the OOB classification error rate of these *OAMs* for predicting HCC is greater than or equal to 0.6, which seemed to have the lowest predictive power for HCC.

Finally, we extracted the 11 highly correlated gene pairs (involving 15 genes in total) from the 12 *OAMs* (OOB classification error rate < 0.5), which met the following two conditions: the top 5 important genes in the *OAMs* according to the MDG, and significantly correlated gene pairs (r > 0.8, P-value < 0.001), as listed in column 7 of Additional file 1: Table S5.

#### Associations of the 11 highly correlated gene pairs in the three disease states

Next, the Pearson correlation coefficients between the 11 pairs of genes in CHB, cirrhosis, and HCC were calculated. The 11 gene pairs were correlated in the three disease states (r > 0.63) (Fig. 5e–h). Furthermore, the changing trends in the correlation of 6 gene pairs in the three disease states were consistent with the disease states indicated by the *OAMs* that the gene pairs belong to (Fig. 5h). The changing trends were roughly divided into the following four categories. (1) Weak correlation with CHB but strong correlation with cirrhosis and HCC. The correlation coefficient of diablo-ebp was 0.72 in CHB and increased to 0.89 and 0.9 in cirrhosis and HCC, respectively. (2) Strong correlation with CHB but weak correlation with cirrhosis and HCC. The correlation of decr1-pik3ca and tnfrsf10b-ebp in CHB was 0.95 and 0.96, respectively, while it decreased in both cirrhosis and HCC. (3) Correlation with cirrhosis different from that with CHB and HCC. The correlation of mgmt-socs1 was 0.96 in CHB but reduced to 0.68 in cirrhosis and then increased to 0.92 in HCC. (4) Strong correlation with CHB, cirrhosis and HCC. The gene pair hdac2-prkaa1 was highly correlated in the three disease states, in accordance with the disease states indicated by *AM*^*O*^_*CHB 23-C11-HCC38*_ (Fig. 5h).

Furthermore, 10 of the 15 genes have been previously reported to be associated with the disease states represented by their *OAMs*, except that *decr1*, *mgmt, diablo* and *ebp* have not been reported to be associated with CHB and *hdac2* has not been reported to be correlated with cirrhosis and HCC (Additional file 1: Table S6). Moreover, 9 of the 15 genes (60%) have been previously reported as biomarkers of HCC (Additional file 1: Table S7).

### Assessing the predictive performance of the 15 genes for HCC using the TCGA LIHC dataset

#### Predictive performance of the 15-gene set

**Fig. 6:**
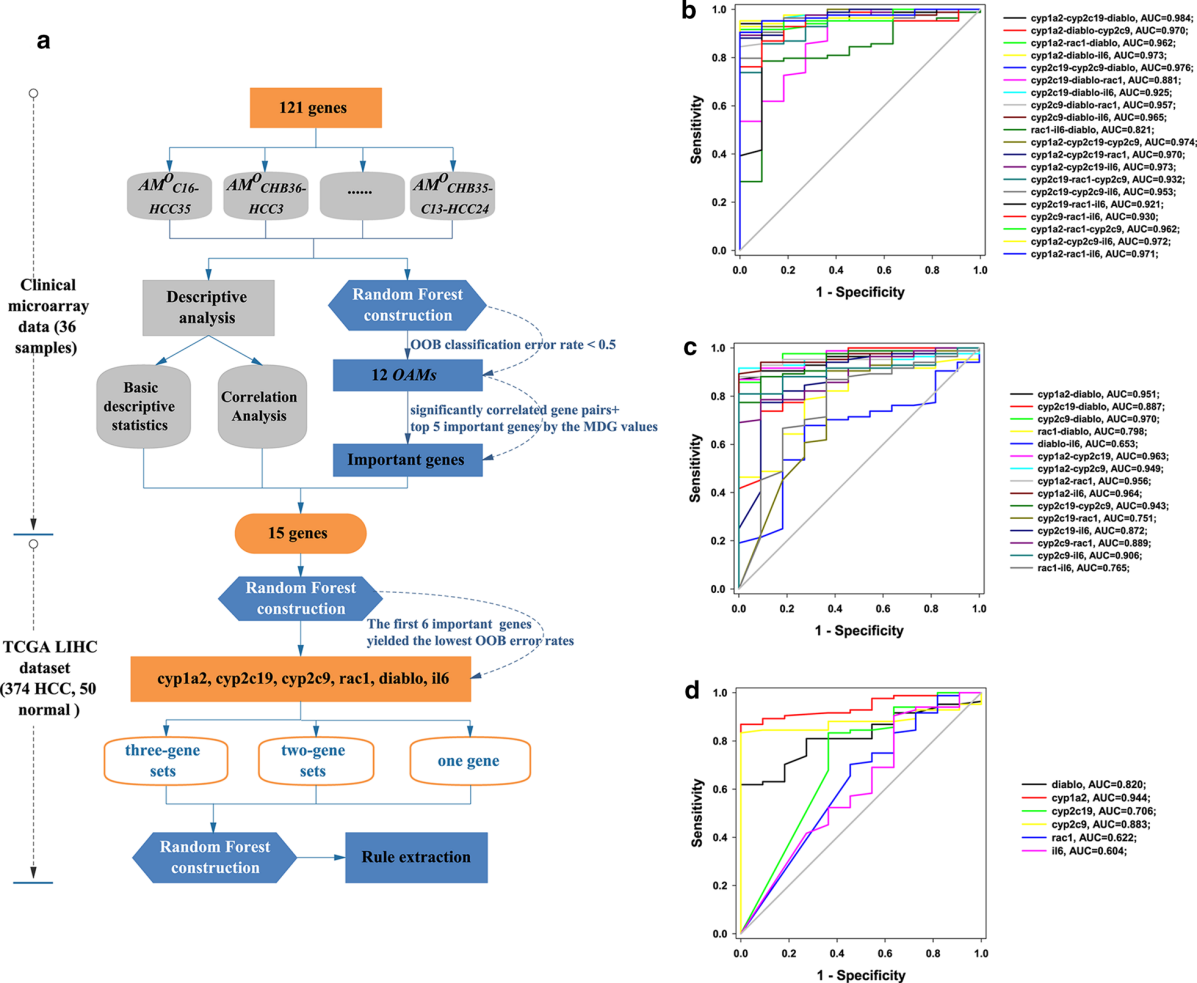
The Random Forest construction and receiver operating characteristic (ROC) curve of candidate genes. **a** The flow chart of Random Forest construction. ROC curve for the relative expression of HCC (n = 84) and non-tumor (n = 11) mRNA-seq samples of each validated gene and gene combination. The corresponding area under the curve (AUC) value is indicated. Diagonal lines represent the performance of a random classifier. **b** ROC curves of three-gene sets for classifying non-tumor samples from HCC samples in the TCGA test set. All three-gene sets achieved an AUC > 0.82. **c** ROC curves of two-gene sets for classifying non-tumor samples from HCC samples in the TCGA test set. All two-gene sets achieved an AUC > 0.65. **d** ROC curves of 6 candidate genes for classifying non-tumor samples from HCC samples in the TCGA test set. All 6 genes achieved an AUC > 0.6

The 15 genes were further evaluated to distinguish tumor tissues from non-tumor tissues by using the TCGA LIHC dataset. The training and test sets were randomly sampled at a 4:1 ratio, with 329 and 95 samples. The random forests algorithm was used to construct a predictive model for HCC in the training sets. The flow chart of Random Forest construction is shown in Fig. 6a. The results showed the classification evaluation indexes of the model. The total OOB error rate, AUC, G-mean, F-value, sensitivity, precision, specificity, and accuracy were 7.6%, 0.99, 0.8991, 0.9823, 0.9881, 0.9765, 0.8182, and 0.9684, respectively.

#### Predictive performance of three-gene sets, two-gene sets, and one gene

The importance of the 15 genes was evaluated by the MDG values. Starting from 15 genes, the random forests model was constructed for the remaining genes after removing the least important gene in the current model. The results showed that the model of the remaining 6 important genes was the best choice that yielded the lowest OOB error rates (total OOB error rate = 6.69%, OOB classification error rate for predicting HCC = 0.024). In order to further obtain the optimal gene combinations of low dimensions, we selected the combinations of one, two or three genes from the 6 important variables (*cyp1a2, cyp2c19, cyp2c9, rac1, diablo,* and *il6*) to establish prediction models for HCC, that is, we ran the random forests algorithm 41 times. The sensitivities of all gene combinations were above 0.9 (range from 0.9048 to 0.9881). Seventeen gene combinations (9 three-gene sets and 8 two-gene sets) achieved a specificity ≥ 0.6364, and only the three-gene set (cyp1a2-cyp2c19-il6) had a specificity greater than 0.9. Almost all gene combinations achieved an AUC > 0.75 except one gene of *il6*, rac1, cyp2c19, and a two-gene set (diablo-il6). Nineteen gene combinations (14 three-gene sets and 5 two-gene sets) achieved an AUC > 0.95 (Additional file 1: Table S8, Fig. 6b-d).

In summary, the overall predictive performance of all gene combinations was ranked as follows: three-gene sets > two-gene sets > one gene (Additional file 1: Table S8, Fig. 6b–d). All classification evaluation indexes in three-gene combinations were better than those in two-gene combinations. Almost all of the observed differences were statistically significant (P < 0.05), except for G-mean, Precision and Specificity (Additional file 1: Table S8-1). We finally identified a three-gene set (cyp1a2-cyp2c19-il6, total OOB error rates = 5.78%, AUC = 0.9730, G-mean = 0.9305, F-value = 0.9697, sensitivity = 0.9524, precision = 0.9877, specificity = 0.9091, and accuracy = 0.9474) with the optimal predictive performance.

#### Rule extraction for predicting HCC

**Fig. 7:**
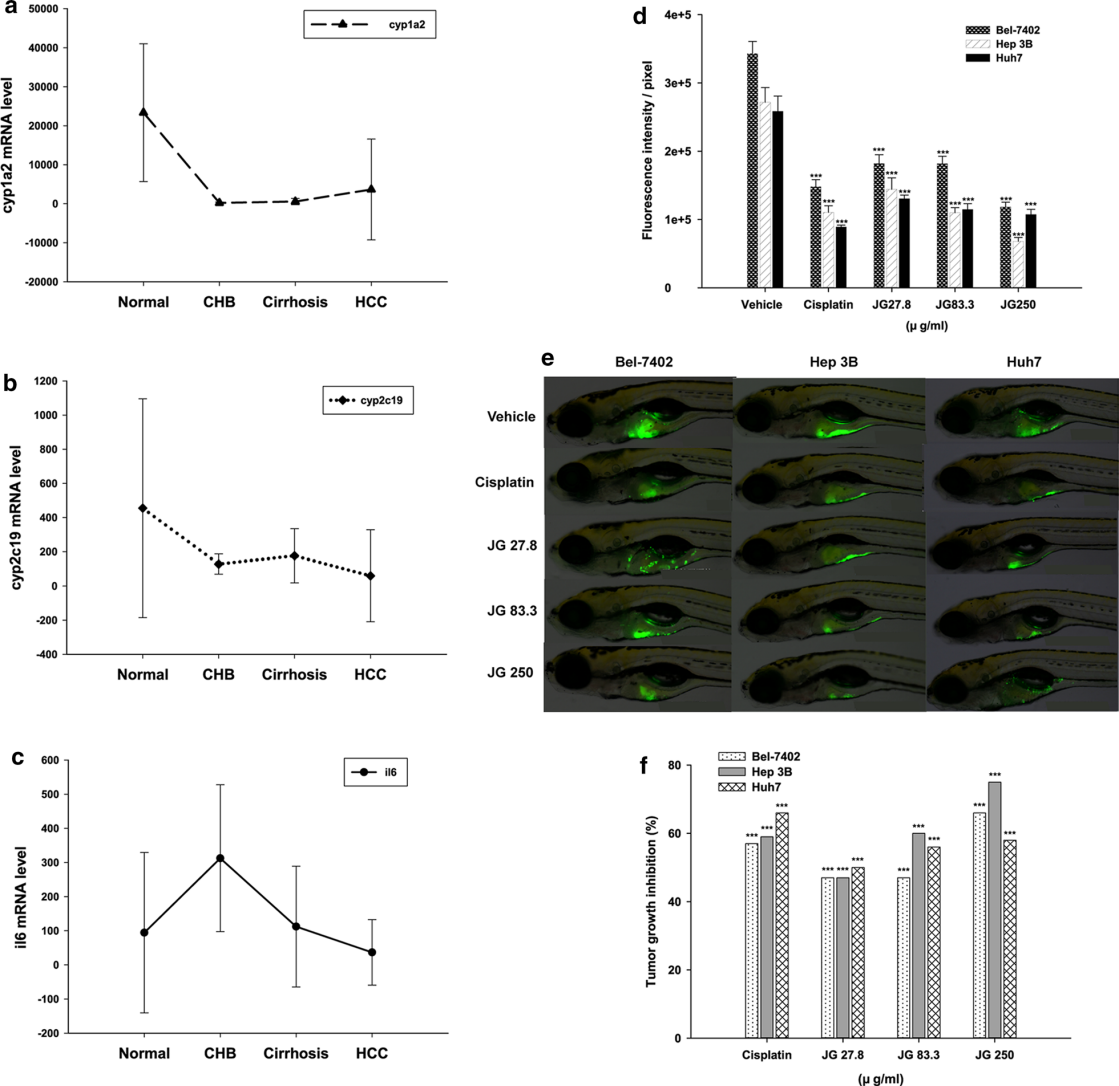
The expression levels of *cyp1a2*, *cyp2c19* and *il6* in different populations and the experimental verification. **a–c** The expression levels of *cyp1a2*, *cyp2c19*, *il6* in different populations. **d**, **e** The fluorescence intensities of different JG granules concentration groups (27.8, 83.3 and 250 µg/mL), the cisplatin group (15 µg/mL) and the vehicle group in Bel-7402, Hep 3B and Huh7 cell lines. ***denotes statistical significance (P-value ≤ 0.001), compared with the vehicle group. **f** The tumor growth inhibition of cisplatin and different JG granules concentration groups (27.8, 83.3 and 250 µg/mL) in Bel-7402, Hep 3B and Huh7 cell lines. ***denotes statistical significance (P-value ≤ 0.001), compared with the vehicle group. *JG *Jiangan granules

Additional file 1: Table S9 shows the 7 most accurate rules. The total error rate was 0.049. The results showed that the present extracted rules achieved a very good performance. Among the 7 conditions, the expression levels of *cyp1a2* and *cyp2c19* in the non-tumor tissues were greater than those in the HCC tissues (Additional file 1: Table S9). The condition “cyp1a2 > 12,201.5 and cyp2c19 <  = 103.5 and il6 <  = 48.5” (error rate = 0.000, frequency = 0.046) might have a greater probability of being correctly predicted as the HCC group. The expression levels of the three genes in different populations (a total of 460 patients, including 53 healthy people, 10 CHB patients, 13 HBV-related cirrhosis patients and 384 HCC patients) were shown in Fig. 7a–c.

### Relationships between gene combinations with good predictive performance and *OAMs*

In combination with the previous *OAMs* results, 80% of the 15 genes were the nodes with the highest degree in the *OAM*s (Additional file 1: Table S10). We found that the two-gene set (cyp1a2-cyp2c19) appeared in the same *OAM* (*AM*^*O*^_*CHB 11-HCC6*_). *Cyp1a2* and *cyp2c19* were located in the overlapping part of *AM*^*O*^_*CHB 11-HCC6*_, and an edge existed between them (Fig. 3b). Both were the nodes with the highest degree (degree = 7, 8) in the module (Additional file 1: Table S10). While *il6* did not appear in the same *OAM* with *cyp1a2* and *cyp2c19*, it appeared in *AM*^*O*^_*CHB*_ _*7-HCC20*_ and *AM*^*O*^_*C2-HCC20*_ separately. It did not have the highest degree (degree = 3) (Additional file 1: Table S10), and it was not located on the overlapping structure of the module (Fig. 3c).

### Experimental verification

The top three compounds that affected the three genes (cyp1a2-cyp2c19-il6) were selected, including glycyrol, inermin and bilobalide (Additional file 1: Table S11). Then, a drug combination (Jiangan granules, JG) containing the three compounds was used to treat three different human HCC cell lines (Bel-7402, Hep 3B and Huh7).

In all three cell lines, the fluorescence intensities of the cisplatin group were significantly reduced compared with the vehicle group (P-value < 0.001) (Fig. 7d, e), and tumor growth inhibition was 57%, 59% and 66%, respectively (Fig. 7f). The fluorescence intensities of different JG granules concentration groups (27.8, 83.3 and 250 µg/mL) were also significantly reduced compared with the vehicle group (P-value ≤ 0.001) (Fig. 7d, e), and tumor growth inhibition was 47%, 47% and 66% (in Bel-7402 cells), 47%, 60% and 75% (in Hep 3B cells), 50%, 56% and 58% (in Huh7 cells), respectively (Fig. 7f). JG granules had a significant inhibitory effect on tumor growth of human HCC transplanted tumors.

## Discussion

The pathogenesis of HCC is complex and heterogenous, and multiple mechanisms of tumorigenesis could be involved. The annual incidence of liver cirrhosis in CHB patients without anti-viral therapy was 2–10%, and the annual incidence of HCC in non-cirrhotic HBV-infected patients was 0.5–1.0%. The annual incidence of HCC in patients with cirrhosis was 3–6% [28]. Despite a large number of promising molecules, the heterogeneity of HCC makes early detection a major challenge [29, 30], and individual markers generally lack sensitivity and/or specificity to be sufficiently effective. The future of HCC screening will most likely involve the use of a combination of biomarkers based on various macromolecules such as mRNAs, proteins, and miRNAs [31].

### Sequential *AMs* contributed to revealing the dynamic evolution from CHB to cirrhosis and HCC

The clinical pathway of most HBV-related HCC may follow the four states: healthy, hepatitis, cirrhosis, and HCC. In our study, the cohort included healthy individuals and patients with CHB, HBV-related cirrhosis and HCC. Using the *AMs*-based approach, four types of modular allostery (*DEMs*, *CAMs*, *TAMs* and *OAMs*) were identified that might reveal the dynamic evolution of pathological processes from CHB to HCC. Module-module associations (finally forming the *AM*s) among CHB, cirrhosis and HCC were established through the partially overlapping structures, which were similar to the linkers connecting domains in protein allostery, implying topological variations in modular networks. Identification of 13 potential *OAMs* also reflected three disease processes in HBV-related HCC cases: from HBV to cirrhosis to HCC, from cirrhosis to HCC, and from HBV to HCC directly. It was also consistent with previous findings that not all patients with HCC have underlying liver cirrhosis, especially CHB patients [32]. The *OAMs* were the partially overlapping modules among different stages in the progression of chronic liver diseases. At different stages, the structures and functions of these modules have partial differences, and further changes may occur.

In addition, the invariant modules *CAMs* might reflect the conservation and stability of the organism. As for *DEMs*, they were the differential modules only found in the three diseases, representing the feature modules unique to CHB, HBV-related cirrhosis or HCC. We identified 35, 6, and 44 *DEM*s in the CHB, cirrhosis, and HCC groups, respectively. *DEMs* might demonstrate the unique characteristics of each stage of hepatitis, cirrhosis and liver cancer. From the perspective of Modular Pharmacology, sequential *AMs* might contribute to illustrating the molecular mechanism of the pathological progression from CHB to HCC. *CAMs*, *OAMs* and *DEMs* might have pharmacological implications at the systems level and serve as universal or specific therapeutic targets in disease treatment [33, 34]. Further, *OAMs* might play an important role in the pathological progression from CHB to cirrhosis to HCC, and therefore had considerable clinical value in predicting early-stage HCC risk.

### Functional changes of *OAMs*: alterations in multiple cellular signaling pathways

As shown in Fig. 4c, d, the carcinogenic effects of the 13 *OAM*s involve different changes in multiple signaling pathways at different pathological stages. We infer that alterations in these signaling pathways as well as some molecular targets in the pathways might participate in critical steps in the development of HBV-associated HCC. The most frequent pathway, the neurotrophin signaling pathway, appeared in four *OAMs*, showing that the dysregulation of neurotrophin signaling might play a role in the progression of HCC [35]. Evidence indicates that growth factor-mediated angiogenic signaling (VEGF, EGFR, IGF and HGF/c-MET), the ERK/MAPK pathway, the PI3K–AKT–mTOR signaling pathway, the WNT/b-catenin pathway, cytokine/chemokine production/activation, leukocyte infiltration, c-erbB-3, adherens junction, focal adhesion, and antigen processing and presentation are implicated in HCC [36–43]. In the erbB family, upregulated ERBB-2 was associated with HBV infection [44]. HBV alters TLR signaling, resulting in liver damage [45]. NK cells are important in the defense against HBV infection and exert their antiviral functions and host anticancer defense by natural cytotoxicity [46, 47]. In addition, *AM*^*O*^_*CHB11-HCC6*_, which is only enriched in 6 metabolism pathways, might be a metabolism-related module. Aberrations in lipid metabolism are often seen in chronic HBV infection. Downregulated linoleic acid [48], increased arachidonic acid synthesis [48] and high serum levels of retinol [49] and cytochrome P450 enzyme [50] are involved in the development of HCC.

### Establishing a panel of genes to predict HCC risk for patients with chronic liver disease

In this study, 11 pairs of highly correlated genes and a panel of genes (cyp1a2-cyp2c19-il6) were identified in the core *OAMs* throughout the progression of CHB to cirrhosis and HCC. Almost all gene combinations achieved an AUC > 0.75. Generally, a larger AUC value indicates a better predictive model and is a commonly accepted rule in the determination of a model’s performance [51]. A classification model can be considered to have an outstanding performance if the AUC value of the model is above 0.9. The performance of any classification model with AUC values between 0.8 and 0.9 is excellent [52]. Therefore, this result indicated that the 6 important genes and their combinations were successfully validated in the independent TCGA LIHC dataset and were able to accurately distinguish HCC from non-tumor tissues. A gene with an AUC value of at least 0.95 and a sensitivity and specificity of 90% or greater at the established threshold is considered adequate for the confident identification of HCC samples [31]. In addition to these criteria, we considered multiple indexes (total OOB error rates, G-mean, F-value, sensitivity, precision and specificity) as a whole and finally identified a three-gene set (cyp1a2-cyp2c19-il6) with an AUC of 0.973, a sensitivity of 0.9524, and a specificity of 0.9091. Here, considering the imbalance of the data, we mainly refer to total OOB error rates, AUC, G-mean and F-value. We also extracted the 7 most accurate rules/conditions from random forests for the three genes (*cyp1a2*, *cyp2c19* and *il6*). Furthermore, the three genes have been previously reported to be associated with HCC [31, 53, 54], which is consistent with the results of rule extraction.

In addition, the results of experimental verification indicated that JG granules had a significant inhibitory effect on tumor growth of human HCC transplanted tumors. JG granules was the drug combination containing the three compounds selected by the three genes (*cyp1a2, cyp2c19* and *il6*), which could indirectly validate the effect of the three genes on the development of HCC. Furthermore, the two-gene set (cyp1a2-cyp2c19, AUC = 0.963) appeared in the same *OAM* (*AM*^*O*^_*CHB11-HCC6*_), *cyp1a2* and *cyp2c19* had the highest within-module degree, and an edge existed between them. This finding also confirmed the reliability of the *AMs*-based approach.

Finally, the limitation of this study is the lack of independent validation. In order to improve the accuracy of prediction, next we will validate the sensitivity and specificity of the three-gene set identified in our study by using an independent, large and multicenter cohort, and furtherly evaluate the diagnostic performance of the three-gene set in different Barcelona Clinic Liver Cancer (BCLC) stages. In addition, the performance of the three-gene set in differentiating the HCC group from the healthy, CHB, and cirrhosis groups will be also evaluated.

## Conclusions

Taken together, we showed that the three-gene set (cyp1a2-cyp2c19-il6) was optimized to distinguish HCC from non-tumor samples using random forests with an AUC of 0.973. These findings indicated that the proposed sequential *AMs*-based approach contributed to revealing the dynamic evolution from CHB to cirrhosis and HCC, identifying a panel of genes for the assessment of HCC risk in patients with chronic liver disease and might be applied to any time-dependent cancer risk prediction.

## Supplementary Information

**Additional file 1: Table S1.** Topological attributes of disease-associated networks and identified functional modules according to the minimum entropy criterion. **Table S2.** MCODE results for CHB-associated networks, cirrhosis-associated networks, and HCC-associated networks. **Table S3.** The number of overlapping pathways among CHB, cirrhosis and HCC in 13 *OAMs*. **Table S4.** Relationships between 24 Altered Pathways/overlapping pathways and CHB/cirrhosis/HCC supported by previous literature. **Table S5.** Highly correlated gene pairs and top 5 important genes in the 13 *OAMs.* **Table S6.** The associations between highly correlated genes in 13 *OAMs* and three diseases. **Table S7.** The relationships between 15 genes and HCC biomarkers in literature. **Table S8.** The classification evaluation indexes of candidate genes and gene combinations for HCC identification. **Table S9.** The 7 most accurate rules and intergroup comparisons of *cyp1a2*, *cyp2c19* and *il6*. **Table S10.** Topological parameters of the 15 genes located in the *OAMs*. **Table S11.** The top three compounds that affected the three genes (*cyp1a2, cyp2c19* and *il6*).

## Data Availability

The datasets used and/or analysed during the current study are available from the corresponding author on reasonable request.

## Abbreviations

AMs: Allosteric modules
AUC: Area under the curve
CHB: Chronic hepatitis B
CAMs: Conserved allosteric modules
DEMs: Disease-exclusive modules
LIHC: Liver hepatocellular carcinoma
HBV: Hepatitis B virus
HCC: Hepatocellular carcinoma
MP: Modular pharmacology
OAMs: Oncogenic allosteric modules
OOB: Out-of-bag
ROC curve: Receiver operating characteristic curve
TCGA: The Cancer Genome Atlas
TAMs: Transitional allosteric modules
