# Supplementary material for: Prediction of hepatocellular carcinoma risk in patients with chronic liver disease from dynamic modular networks
Source: J Transl Med. 2021 Mar 23;19:122. doi: 10.1186/s12967-021-02791-9 (PMC7989040; doi:10.1186/s12967-021-02791-9)
Supplement: Supplementary file 1 — Additional file 1: Table S1. Topological attributes of disease-associated networks and identified functional modules according to the minimum entropy criterion. Table S2. MCODE results for CHB-associated networks, cirrhosis-associated networks, and HCC-associated networks. Table S3. The number of overlapping pathways among CHB, cirrhosis and HCC in 13 OAMs. Table S4. Relationships between 24 Altered Pathways/overlapping pathways and CHB/cirrhosis/HCC supported by previous literature. Table S5. Highly correlated gene pairs and top 5 important genes in the 13 OAMs. Table S6. The associations between highly correlated genes in 13 OAMs and three diseases. Table S7. The relationships between 15 genes and HCC biomarkers in literature. Table S8. The classification evaluation indexes of candidate genes and gene combinations for HCC identification. Table S9. The 7 most accurate rules and intergroup comparisons of cyp1a2, cyp2c19 and il6. Table S10. Topological parameters of the 15 genes located in the OAMs. Table S11. The top three compounds that affected the three genes (cyp1a2, cyp2c19 and il6). [file 12967_2021_2791_MOESM1_ESM.docx]

**Additional file 1**

**Table S1.** Topological attributes of disease-associated networks and identified functional modules according to the minimum entropy criterion.

**Table S2.** MCODE results for CHB-associated networks, cirrhosis-associated networks, and HCC-associated networks.

**Table S3.** The number of overlapping pathways among CHB, cirrhosis and HCC in 13 *OAMs*.

**Table S4.** Relationships between 24 Altered Pathways/overlapping pathways and CHB/cirrhosis/HCC supported by previous literature.

**Table S5.** Highly correlated gene pairs and top 5 important genes in the 13 *OAMs.*

**Table S6.** The associations between highly correlated genes in 13 *OAMs* and three diseases.

**Table S7.** The relationships between 15 genes and HCC biomarkers in literature.

**Table S8.** The classification evaluation indexes of candidate genes and gene combinations for HCC identification.

**Table S9.** The 7 most accurate rules and intergroup comparisons of *cyp1a2*, *cyp2c19* and *il6*.

**Table S10.** Topological parameters of the 15 genes located in the *OAMs*.

**Table S11.** The top three compounds that affected the three genes (*cyp1a2, cyp2c19* and *il6*).

**Table S1 Topological attributes of disease-associated networks and identified functional modules according to the minimum entropy criterion**

| **Disease stages** | **OMIM terms** | **Networks** | | **Search terms** | |
| --- | --- | --- | --- | --- | --- |
|  |  | **Nodes** | **Edges** |  |  |
| **CHB** | 220 | 1104 | 3421 | 103 | |
| **Cirrhosis** | 152 | 487 | 1131 | 42 | |
| **HCC** | 213 | 1079 | 3085 | 98 | |
|  | | | | | |
| **Identified functional modules** | | | | | |
| **Disease stages** | - **Number of modules**   **（N≥4）** | **Average size** | **Maximum size** | **Modularity** | **Entropy** |
| **CHB** | 53 | 5.163 | 17 | 0.314 | 5.91751 |
| **Cirrhosis** | 21 | 4.609 | 15 | 0.404 | 5.13214 |
| **HCC** | 60 | 5.233 | 19 | 0.405 | 5.9334 |

**Table S2 MCODE results for CHB-associated networks, cirrhosis-associated networks, and HCC-associated networks**

| **CHB-associated networks** | | | | | | |
| --- | --- | --- | --- | --- | --- | --- |
| **Parameters** | **Clusters** | **Average size** | **Maximum size** | **Minimum size** | **Modularity** | **Entropy** |
| 1 | 77 | 6.766 | 57 | 3 | 0.304 | 6.04314 |
| 2 | 86 | 6.047 | 38 | 3 | 0.331 | 6.04487 |
| 3 | 90 | 5.767 | 30 | 3 | 0.33 | 6.06369 |
| 4 | 89 | 5.787 | 24 | 3 | 0.346 | 6.05612 |
| 5 | 77 | 20.13 | 254 | 3 | 0.556 | 6.47849 |
| 6 | 86 | 18.442 | 229 | 3 | 0.684 | 6.46949 |
| 7 | 90 | 17.778 | 230 | 3 | 0.723 | 6.4915 |
| 8 | 89 | 18.27 | 180 | 3 | 0.758 | 6.5005 |
| 9 | 77 | 7.039 | 57 | 3 | 0.309 | 6.26816 |
| 10 | 86 | 6.337 | 39 | 3 | 0.333 | 6.25453 |
| 11 | 90 | 6 | 30 | 3 | 0.334 | 6.28159 |
| 12 | 89 | 6 | 24 | 3 | 0.35 | 6.26728 |
| 13 | 77 | 20.701 | 254 | 3 | 0.571 | 6.49511 |
| 14 | 86 | 19.523 | 244 | 3 | 0.71 | 6.484 |
| 15 | 90 | 18.556 | 230 | 3 | 0.748 | 6.51749 |
| 16 | 89 | 19.056 | 180 | 3 | 0.786 | 6.51785 |
| 17 | 82 | 5.5 | 17 | 3 | 0.315 | 6.34766 |
| 18 | 84 | 5.357 | 17 | 3 | 0.314 | 6.34575 |
| 19 | 85 | 5.294 | 17 | 3 | 0.315 | 5.91895 |
| 20 | 86 | 5.163 | 17 | 3 | 0.314 | 5.91751 |
| 21 | 82 | 18.305 | 144 | 3 | 0.755 | 6.42498 |
| 22 | 84 | 17.869 | 144 | 3 | 0.754 | 6.4217 |
| 23 | 85 | 17.753 | 144 | 3 | 0.761 | 6.4229 |
| 24 | 86 | 16.686 | 144 | 3 | 0.73 | 6.418 |
| 25 | 82 | 5.61 | 17 | 3 | 0.318 | 6.18605 |
| 26 | 84 | 5.476 | 17 | 3 | 0.317 | 6.1912 |
| 27 | 85 | 5.376 | 17 | 3 | 0.316 | 6.18215 |
| 28 | 86 | 5.244 | 17 | 3 | 0.314 | 6.19141 |
| 29 | 82 | 18.61 | 144 | 3 | 0.765 | 6.433 |
| 30 | 84 | 18.25 | 144 | 3 | 0.769 | 6.43209 |
| 31 | 85 | 18.047 | 144 | 3 | 0.769 | 6.42662 |
| 32 | 86 | 17.674 | 144 | 3 | 0.768 | 6.42466 |
| 33 | 57 | 9.404 | 98 | 3 | 0.295 | 6.22572 |
| 34 | 76 | 7.013 | 49 | 3 | 0.326 | 6.22661 |
| 35 | 75 | 7.013 | 39 | 3 | 0.333 | 6.23355 |
| 36 | 82 | 6.39 | 24 | 3 | 0.351 | 6.23808 |
| 37 | 57 | 25.14 | 310 | 3 | 0.449 | 6.47966 |
| 38 | 76 | 20.329 | 291 | 3 | 0.658 | 6.48296 |
| 39 | 75 | 20.267 | 291 | 3 | 0.654 | 6.48258 |
| 40 | 82 | 20.073 | 211 | 3 | 0.7 | 6.48738 |
| 41 | 57 | 9.965 | 102 | 3 | 0.3 | 6.24728 |
| 42 | 76 | 7.447 | 52 | 3 | 0.333 | 6.2649 |
| 43 | 81 | 5.654 | 32 | 3 | 0.753 | 6.24336 |
| 44 | 82 | 6.695 | 24 | 3 | 0.357 | 6.26265 |
| 45 | 57 | 26.193 | 310 | 3 | 0.466 | 6.49894 |
| 46 | 76 | 21.382 | 291 | 3 | 0.687 | 6.50006 |
| 47 | 75 | 21.507 | 291 | 3 | 0.691 | 6.50214 |
| 48 | 82 | 21.061 | 211 | 3 | 0.727 | 6.50949 |
|  |  |  |  |  |  |  |
|  |  |  |  |  |  |  |
| **Cirrhosis-associated networks** | | | | | | |
| **Parameters** | **Clusters** | **Average size** | **Maximum size** | **Minimum size** | **Modularity** | **Entropy** |
| 1 | 45 | 5.089 | 24 | 3 | 0.451 | 5.17551 |
| 2 | 48 | 4.792 | 24 | 3 | 0.463 | 5.17844 |
| 3 | 47 | 4.851 | 24 | 3 | 0.463 | 5.18548 |
| 4 | 48 | 4.729 | 24 | 3 | 0.46 | 5.17706 |
| 5 | 45 | 10.333 | 75 | 3 | 0.705 | 5.50755 |
| 6 | 48 | 9.938 | 66 | 3 | 0.735 | 5.51066 |
| 7 | 47 | 10.17 | 66 | 3 | 0.742 | 5.51701 |
| 8 | 48 | 9.812 | 66 | 3 | 0.72 | 5.51066 |
| 9 | 45 | 5.222 | 24 | 3 | 0.452 | 5.3167 |
| 10 | 48 | 4.896 | 24 | 3 | 0.466 | 5.33638 |
| 11 | 47 | 4.957 | 24 | 3 | 0.466 | 5.33708 |
| 12 | 48 | 4.854 | 24 | 3 | 0.464 | 5.33638 |
| 13 | 45 | 10.911 | 75 | 3 | 0.731 | 5.52845 |
| 14 | 48 | 10.333 | 66 | 3 | 0.761 | 5.52569 |
| 15 | 47 | 10.489 | 66 | 3 | 0.76 | 5.53093 |
| 16 | 48 | 10.271 | 66 | 3 | 0.758 | 5.52569 |
| 17 | 45 | 4.622 | 20 | 3 | 0.395 | 5.41228 |
| 18 | 46 | 4.522 | 15 | 3 | 0.403 | 5.42221 |
| 19 | 46 | 4.522 | 15 | 3 | 0.403 | 5.42221 |
| 20 | 46 | 4.522 | 15 | 3 | 0.403 | 5.42221 |
| 21 | 45 | 9.578 | 75 | 3 | 0.673 | 5.53601 |
| 22 | 46 | 9.413 | 66 | 3 | 0.691 | 5.5387 |
| 23 | 46 | 9.413 | 66 | 3 | 0.691 | 5.5387 |
| 24 | 46 | 9.435 | 66 | 3 | 0.693 | 5.5351 |
| 25 | 45 | 4.711 | 20 | 3 | 0.396 | 5.37134 |
| 26 | 46 | 4.609 | 15 | 3 | 0.404 | 5.13214 |
| 27 | 46 | 4.587 | 15 | 3 | 0.403 | 5.13214 |
| 28 | 46 | 4.565 | 15 | 3 | 0.404 | 5.13214 |
| 29 | 45 | 10.067 | 75 | 3 | 0.701 | 5.5033 |
| 30 | 46 | 9.891 | 66 | 3 | 0.718 | 5.50652 |
| 31 | 46 | 9.848 | 66 | 3 | 0.716 | 5.50652 |
| 32 | 46 | 9.609 | 66 | 3 | 0.701 | 5.50652 |
| 33 | 39 | 6.051 | 26 | 3 | 0.479 | 5.33745 |
| 34 | 42 | 5.595 | 24 | 3 | 0.49 | 5.34779 |
| 35 | 42 | 5.5 | 24 | 3 | 0.478 | 5.34703 |
| 36 | 43 | 5.349 | 24 | 3 | 0.472 | 5.31528 |
| 37 | 39 | 11.564 | 104 | 3 | 0.666 | 5.52528 |
| 38 | 42 | 10.905 | 95 | 3 | 0.695 | 5.52701 |
| 39 | 42 | 11 | 95 | 3 | 0.701 | 5.52917 |
| 40 | 43 | 10.744 | 95 | 3 | 0.707 | 5.51581 |
| 41 | 39 | 6.179 | 26 | 3 | 0.479 | 5.34086 |
| 42 | 42 | 5.667 | 24 | 3 | 0.492 | 5.3502 |
| 43 | 42 | 5.571 | 24 | 3 | 0.48 | 5.31821 |
| 44 | 43 | 5.465 | 24 | 3 | 0.474 | 5.32398 |
| 45 | 39 | 11.949 | 104 | 3 | 0.676 | 5.53237 |
| 46 | 42 | 11.167 | 95 | 3 | 0.71 | 5.53179 |
| 47 | 42 | 11.167 | 95 | 3 | 0.71 | 5.53356 |
| 48 | 43 | 11.209 | 95 | 3 | 0.733 | 5.52613 |
|  |  |  |  |  |  |  |
|  |  |  |  |  |  |  |
| **HCC-associated networks** | | | | | | |
| **Parameters** | **Clusters** | **Average size** | **Maximum size** | **Minimum size** | **Modularity** | **Entropy** |
| 1 | 70 | 7.429 | 90 | 3 | 0.39 | 6.02628 |
| 2 | 83 | 6.193 | 45 | 3 | 0.425 | 6.02576 |
| 3 | 86 | 5.872 | 37 | 3 | 0.419 | 6.01659 |
| 4 | 90 | 5.656 | 19 | 3 | 0.423 | 6.01462 |
| 5 | 70 | 17.457 | 307 | 3 | 0.618 | 6.39403 |
| 6 | 83 | 15.277 | 203 | 3 | 0.749 | 6.39823 |
| 7 | 86 | 14.535 | 191 | 3 | 0.771 | 6.38124 |
| 8 | 90 | 14.567 | 104 | 3 | 0.819 | 6.39268 |
| 9 | 70 | 7.7 | 91 | 3 | 0.393 | 6.17646 |
| 10 | 83 | 6.434 | 45 | 3 | 0.428 | 6.17721 |
| 11 | 86 | 6.151 | 37 | 3 | 0.423 | 6.02284 |
| 12 | 90 | 5.833 | 19 | 3 | 0.426 | 6.03321 |
| 13 | 70 | 17.986 | 308 | 3 | 0.628 | 6.408 |
| 14 | 83 | 15.952 | 203 | 3 | 0.767 | 6.40068 |
| 15 | 86 | 15.395 | 191 | 3 | 0.795 | 6.39129 |
| 16 | 90 | 15.233 | 104 | 3 | 0.84 | 6.40582 |
| 17 | 85 | 5.518 | 24 | 3 | 0.401 | 6.23109 |
| 18 | 88 | 5.307 | 19 | 3 | 0.407 | 6.23743 |
| 19 | 90 | 5.144 | 19 | 3 | 0.403 | 6.23928 |
| 20 | 91 | 5.132 | 19 | 3 | 0.408 | 6.23331 |
| 21 | 85 | 14.141 | 162 | 3 | 0.733 | 6.37504 |
| 22 | 88 | 13.773 | 116 | 3 | 0.784 | 6.36993 |
| 23 | 90 | 13.278 | 113 | 3 | 0.78 | 6.36653 |
| 24 | 91 | 13.484 | 103 | 3 | 0.803 | 6.36504 |
| 25 | 85 | 5.565 | 24 | 3 | 0.403 | 6.15003 |
| 26 | 88 | 5.375 | 19 | 3 | 0.408 | 5.93848 |
| 27 | 90 | 5.233 | 19 | 3 | 0.405 | 5.9334 |
| 28 | 91 | 5.176 | 19 | 3 | 0.409 | 5.93408 |
| 29 | 85 | 14.306 | 162 | 3 | 0.741 | 6.35994 |
| 30 | 88 | 14 | 116 | 3 | 0.791 | 6.35643 |
| 31 | 90 | 13.744 | 113 | 3 | 0.795 | 6.35175 |
| 32 | 91 | 13.659 | 103 | 3 | 0.812 | 6.3466 |
| 33 | 66 | 8.227 | 79 | 3 | 0.416 | 6.19164 |
| 34 | 77 | 7.078 | 49 | 3 | 0.449 | 6.19785 |
| 35 | 80 | 6.762 | 37 | 3 | 0.445 | 6.19268 |
| 36 | 87 | 6.23 | 21 | 3 | 0.453 | 6.19814 |
| 37 | 66 | 19.061 | 275 | 3 | 0.643 | 6.40766 |
| 38 | 77 | 16.701 | 213 | 3 | 0.755 | 6.41506 |
| 39 | 80 | 16.3 | 191 | 3 | 0.796 | 6.41688 |
| 40 | 87 | 15 | 122 | 3 | 0.829 | 6.4294 |
| 41 | 66 | 8.606 | 79 | 3 | 0.419 | 6.2261 |
| 42 | 77 | 7.377 | 49 | 3 | 0.454 | 6.23531 |
| 43 | 80 | 7.075 | 37 | 3 | 0.451 | 6.21427 |
| 44 | 87 | 6.483 | 26 | 3 | 0.456 | 6.22107 |
| 45 | 66 | 20.03 | 275 | 3 | 0.664 | 6.43764 |
| 46 | 77 | 17.377 | 213 | 3 | 0.773 | 6.43977 |
| 47 | 80 | 17.363 | 191 | 3 | 0.827 | 6.43426 |
| 48 | 87 | 16.333 | 122 | 3 | 0.862 | 6.43855 |

**MCODE parameter settings**

In the MCODE algorithm, we tried all the possible combinations of the key parameters:

- Parameters 1: Include Loops: false; Degree Cutoff: 3; Node Score Cutoff: 0.2; Haircut: true; Fluff: false; K-Core: 2; Max. Depth from Seed: 100
- Parameters 2: Include Loops: false; Degree Cutoff: 3; Node Score Cutoff: 0.2; Haircut: true; Fluff: false; K-Core: 2; Max. Depth from Seed: 5
- Parameters 3: Include Loops: false; Degree Cutoff: 3; Node Score Cutoff: 0.2; Haircut: true; Fluff: false; K-Core: 2; Max. Depth from Seed: 4
- Parameters 4: Include Loops: false; Degree Cutoff: 3; Node Score Cutoff: 0.2; Haircut: true; Fluff: false; K-Core: 2; Max. Depth from Seed: 3
- Parameters 5: Include Loops: false; Degree Cutoff: 3; Node Score Cutoff: 0.2; Haircut: true; Fluff: true; Fluff Density Cutoff 0.1; K-Core: 2; Max. Depth from Seed: 100
- Parameters 6: Include Loops: false; Degree Cutoff: 3; Node Score Cutoff: 0.2; Haircut: true; Fluff: true; Fluff Density Cutoff 0.1; K-Core: 2; Max. Depth from Seed: 5
- Parameters 7: Include Loops: false; Degree Cutoff: 3; Node Score Cutoff: 0.2; Haircut: true; Fluff: true; Fluff Density Cutoff 0.1; K-Core: 2; Max. Depth from Seed: 4
- Parameters 8: Include Loops: false; Degree Cutoff: 3; Node Score Cutoff: 0.2; Haircut: true; Fluff: true; Fluff Density Cutoff 0.1; K-Core: 2; Max. Depth from Seed: 3
- Parameters 9: Include Loops: false; Degree Cutoff: 3; Node Score Cutoff: 0.2; Haircut: false; Fluff: false; K-Core: 2; Max. Depth from Seed: 100
- Parameters 10: Include Loops: false; Degree Cutoff: 3; Node Score Cutoff: 0.2; Haircut: false; Fluff: false; K-Core: 2; Max. Depth from Seed: 5
- Parameters 11: Include Loops: false; Degree Cutoff: 3; Node Score Cutoff: 0.2; Haircut: false; Fluff: false; K-Core: 2; Max. Depth from Seed: 4
- Parameters 12: Include Loops: false; Degree Cutoff: 3; Node Score Cutoff: 0.2; Haircut: false; Fluff: false; K-Core: 2; Max. Depth from Seed: 3
- Parameters 13: Include Loops: false; Degree Cutoff: 3; Node Score Cutoff: 0.2; Haircut: false; Fluff: true; K-Core: 2; Max. Depth from Seed: 100
- Parameters 14: Include Loops: false; Degree Cutoff: 3; Node Score Cutoff: 0.2; Haircut: false; Fluff: true; K-Core: 2; Max. Depth from Seed: 5
- Parameters 15: Include Loops: false; Degree Cutoff: 3; Node Score Cutoff: 0.2; Haircut: false; Fluff: true; K-Core: 2; Max. Depth from Seed: 4
- Parameters 16: Include Loops: false; Degree Cutoff: 3; Node Score Cutoff: 0.2; Haircut: false; Fluff: true; K-Core: 2; Max. Depth from Seed: 3
- Parameters 17: Include Loops: false; Degree Cutoff: 3; Node Score Cutoff: 0.0; Haircut: true; Fluff: false; K-Core: 2; Max. Depth from Seed: 100
- Parameters 18: Include Loops: false; Degree Cutoff: 3; Node Score Cutoff: 0.0; Haircut: true; Fluff: false; K-Core: 2; Max. Depth from Seed: 5
- Parameters 19: Include Loops: false; Degree Cutoff: 3; Node Score Cutoff: 0.0; Haircut: true; Fluff: false; K-Core: 2; Max. Depth from Seed: 4
- Parameters 20: Include Loops: false; Degree Cutoff: 3; Node Score Cutoff: 0.0; Haircut: true; Fluff: false; K-Core: 2; Max. Depth from Seed: 3
- Parameters 21: Include Loops: false; Degree Cutoff: 3; Node Score Cutoff: 0.0; Haircut: true; Fluff: true; Fluff Density Cutoff 0.1; K-Core: 2; Max. Depth from Seed: 100
- Parameters 22: Include Loops: false; Degree Cutoff: 3; Node Score Cutoff: 0.0; Haircut: true; Fluff: true; Fluff Density Cutoff 0.1; K-Core: 2; Max. Depth from Seed: 5
- Parameters 23: Include Loops: false; Degree Cutoff: 3; Node Score Cutoff: 0.0; Haircut: true; Fluff: true; Fluff Density Cutoff 0.1; K-Core: 2; Max. Depth from Seed: 4
- Parameters 24: Include Loops: false; Degree Cutoff: 3; Node Score Cutoff: 0.0; Haircut: true; Fluff: true; Fluff Density Cutoff 0.1; K-Core: 2; Max. Depth from Seed: 3
- Parameters 25: Include Loops: false; Degree Cutoff: 3; Node Score Cutoff: 0.0; Haircut: false; Fluff: false; K-Core: 2; Max. Depth from Seed: 100
- Parameters 26: Include Loops: false; Degree Cutoff: 3; Node Score Cutoff: 0.0; Haircut: false; Fluff: false; K-Core: 2; Max. Depth from Seed: 5
- Parameters 27: Include Loops: false; Degree Cutoff: 3; Node Score Cutoff: 0.0; Haircut: false; Fluff: false; K-Core: 2; Max. Depth from Seed: 4
- Parameters 28: Include Loops: false; Degree Cutoff: 3; Node Score Cutoff: 0.0; Haircut: false; Fluff: false; K-Core: 2; Max. Depth from Seed: 3
- Parameters 29: Include Loops: false; Degree Cutoff: 3; Node Score Cutoff: 0.0; Haircut: false; Fluff: true; K-Core: 2; Max. Depth from Seed: 100
- Parameters 30: Include Loops: false; Degree Cutoff: 3; Node Score Cutoff: 0.0; Haircut: false; Fluff: true; K-Core: 2; Max. Depth from Seed: 5
- Parameters 31: Include Loops: false; Degree Cutoff: 3; Node Score Cutoff: 0.0; Haircut: false; Fluff: true; K-Core: 2; Max. Depth from Seed: 4
- Parameters 32: Include Loops: false; Degree Cutoff: 3; Node Score Cutoff: 0.0; Haircut: false; Fluff: true; K-Core: 2; Max. Depth from Seed: 3
- Parameters 33: Include Loops: false; Degree Cutoff: 3; Node Score Cutoff: 0.3; Haircut: true; Fluff: false; K-Core: 2; Max. Depth from Seed: 100
- Parameters 34: Include Loops: false; Degree Cutoff: 3; Node Score Cutoff: 0.3; Haircut: true; Fluff: false; K-Core: 2; Max. Depth from Seed: 5
- Parameters 35: Include Loops: false; Degree Cutoff: 3; Node Score Cutoff: 0.3; Haircut: true; Fluff: false; K-Core: 2; Max. Depth from Seed: 4
- Parameters 36: Include Loops: false; Degree Cutoff: 3; Node Score Cutoff: 0.3; Haircut: true; Fluff: false; K-Core: 2; Max. Depth from Seed: 3
- Parameters 37: Include Loops: false; Degree Cutoff: 3; Node Score Cutoff: 0.3; Haircut: true; Fluff: true; Fluff Density Cutoff 0.1; K-Core: 2; Max. Depth from Seed: 100
- Parameters 38: Include Loops: false; Degree Cutoff: 3; Node Score Cutoff: 0.3; Haircut: true; Fluff: true; Fluff Density Cutoff 0.1; K-Core: 2; Max. Depth from Seed: 5
- Parameters 39: Include Loops: false; Degree Cutoff: 3; Node Score Cutoff: 0.3; Haircut: true; Fluff: true; Fluff Density Cutoff 0.1; K-Core: 2; Max. Depth from Seed: 4
- Parameters 40: Include Loops: false; Degree Cutoff: 3; Node Score Cutoff: 0.3; Haircut: true; Fluff: true; Fluff Density Cutoff 0.1; K-Core: 2; Max. Depth from Seed: 3
- Parameters 41: Include Loops: false; Degree Cutoff: 3; Node Score Cutoff: 0.3; Haircut: false; Fluff: false; K-Core: 2; Max. Depth from Seed: 100
- Parameters 42: Include Loops: false; Degree Cutoff: 3; Node Score Cutoff: 0.3; Haircut: false; Fluff: false; K-Core: 2; Max. Depth from Seed: 5
- Parameters 43: Include Loops: false; Degree Cutoff: 3; Node Score Cutoff: 0.3; Haircut: false; Fluff: false; K-Core: 2; Max. Depth from Seed: 4
- Parameters 44: Include Loops: false; Degree Cutoff: 3; Node Score Cutoff: 0.3; Haircut: false; Fluff: false; K-Core: 2; Max. Depth from Seed: 3
- Parameters 45: Include Loops: false; Degree Cutoff: 3; Node Score Cutoff: 0.3; Haircut: false; Fluff: true; K-Core: 2; Max. Depth from Seed: 100
- Parameters 46: Include Loops: false; Degree Cutoff: 3; Node Score Cutoff: 0.3; Haircut: false; Fluff: true; K-Core: 2; Max. Depth from Seed: 5
- Parameters 47: Include Loops: false; Degree Cutoff: 3; Node Score Cutoff: 0.3; Haircut: false; Fluff: true; K-Core: 2; Max. Depth from Seed: 4
- Parameters 48: Include Loops: false; Degree Cutoff: 3; Node Score Cutoff: 0.3; Haircut: false; Fluff: true; K-Core: 2; Max. Depth from Seed: 3

**Table S3 The number of overlapping pathways among CHB, cirrhosis and HCC in 13 *OAMs***

| **Groups** | **Overlapping number** | **Overlapping pathways** |
| --- | --- | --- |
| **CHB *vs.* cirrhosis** | 18 | hsa04012:ErbB signaling pathway |
|  |  | hsa04510:Focal adhesion |
|  |  | hsa04520:Adherens junction |
|  |  | hsa04620:Toll-like receptor signaling pathway |
|  |  | hsa04650:Natural killer cell mediated cytotoxicity |
|  |  | hsa04664:Fc epsilon RI signaling pathway |
|  |  | hsa04722:Neurotrophin signaling pathway |
|  |  | hsa04910:Insulin signaling pathway |
|  |  | hsa04914:Progesterone-mediated oocyte maturation |
|  |  | hsa05200:Pathways in cancer |
|  |  | hsa05210:Colorectal cancer |
|  |  | hsa05212:Pancreatic cancer |
|  |  | hsa05213:Endometrial cancer |
|  |  | hsa05214:Glioma |
|  |  | hsa05215:Prostate cancer |
|  |  | hsa05218:Melanoma |
|  |  | hsa05219:Bladder cancer |
|  |  | hsa05222:Small cell lung cancer |
| **CHB *vs.* HCC** | 24 | hsa00232:Caffeine metabolism |
|  |  | hsa00590:Arachidonic acid metabolism |
|  |  | hsa00591:Linoleic acid metabolism |
|  |  | hsa00830:Retinol metabolism |
|  |  | hsa00980:Metabolism of xenobiotics by cytochrome P450 |
|  |  | hsa00982:Drug metabolism |
|  |  | hsa04062:Chemokine signaling pathway |
|  |  | hsa04370:VEGF signaling pathway |
|  |  | hsa04510:Focal adhesion |
|  |  | hsa04612:Antigen processing and presentation |
|  |  | hsa04620:Toll-like receptor signaling pathway |
|  |  | hsa04622:RIG-I-like receptor signaling pathway |
|  |  | hsa04650:Natural killer cell mediated cytotoxicity |
|  |  | hsa04662:B cell receptor signaling pathway |
|  |  | hsa04664:Fc epsilon RI signaling pathway |
|  |  | hsa04666:Fc gamma R-mediated phagocytosis |
|  |  | hsa04670:Leukocyte transendothelial migration |
|  |  | hsa04722:Neurotrophin signaling pathway |
|  |  | hsa04810:Regulation of actin cytoskeleton |
|  |  | hsa05020:Prion diseases |
|  |  | hsa05200:Pathways in cancer |
|  |  | hsa05210:Colorectal cancer |
|  |  | hsa05223:Non-small cell lung cancer |
|  |  | hsa05332:Graft-versus-host disease |
| **HCC *vs.* cirrhosis** | 7 | hsa04510:Focal adhesion |
|  |  | hsa04620:Toll-like receptor signaling pathway |
|  |  | hsa04650:Natural killer cell mediated cytotoxicity |
|  |  | hsa04664:Fc epsilon RI signaling pathway |
|  |  | hsa04722:Neurotrophin signaling pathway |
|  |  | hsa05200:Pathways in cancer |
|  |  | hsa05210:Colorectal cancer |
| **CHB *vs.* cirrhosis *vs.* HCC** | 7 | hsa04510:Focal adhesion |
|  |  | hsa04620:Toll-like receptor signaling pathway |
|  |  | hsa04650:Natural killer cell mediated cytotoxicity |
|  |  | hsa04664:Fc epsilon RI signaling pathway |
|  |  | hsa04722:Neurotrophin signaling pathway |
|  |  | hsa05200:Pathways in cancer |
|  |  | hsa05210:Colorectal cancer |

**Table S4 Relationships between 24 Altered Pathways/overlapping pathways and CHB/cirrhosis/HCC supported by previous literature**

| **Categories** | **Altered pathways** | **Frequency of enrichment** | ***AM*s enriched in the pathways** | **References** | | |
| --- | --- | --- | --- | --- | --- | --- |
|  |  |  |  | **Whether or Not Related to CHB** | **Whether or Not Related to cirrhosis** | **Whether or Not Related to HCC** |
| **Cellular commiunity** | hsa04510:Focal adhesion | 3 | ***AM_C2_***(***AM^O^_C2-HCC20_***) | Yes (1) | not reported | Yes (2) |
|  |  |  | ***AM_HCC3_***(***AM^O^_CHB36-HCC3_***) |  |  |  |
|  |  |  | ***AM_CHB7_***(***AM^O^_CHB7-HCC20_***) |  |  |  |
|  | hsa04520:Adherens junction | 2 | ***AM_CHB5_***(***AM^O^_CHB5-C3-HCC10_***) | Yes (3) | Yes (4) | Yes (4) |
|  |  |  | ***AM_C3_***(***AM^O^_CHB5-C3-HCC10_***) |  |  |  |
| **Immune system** | hsa04664:Fc epsilon RI signaling pathway | 3 | ***AM_C2_***(***AM^O^_C2-HCC20_***) | not reported | not reported | not reported |
|  |  |  | ***AM_HCC3_***(***AM^O^_CHB36-HCC3_***) |  |  |  |
|  |  |  | ***AM_CHB7_***(***AM^O^_CHB7-HCC20_***) |  |  |  |
|  | hsa04662:B cell receptor signaling pathway | 2 | ***AM_HCC3_***(***AM^O^_CHB36-HCC3_***) | not reported | / | not reported |
|  |  |  | ***AM_CHB7_***(***AM^O^_CHB7-HCC20_***) |  |  |  |
|  | hsa04666:Fc gamma R-mediated phagocytosis | 2 | ***AM_HCC3_***(***AM^O^_CHB36-HCC3_***) | not reported | / | not reported |
|  |  |  | ***AM_CHB7_***(***AM^O^_CHB7-HCC20_***) |  |  |  |
|  | hsa04670:Leukocyte transendothelial migration | 2 | ***AM_HCC3_***(***AM^O^_CHB36-HCC3_***) | not reported | / | Yes (5) |
|  |  |  | ***AM_CHB7_***(***AM^O^_CHB7-HCC20_***) |  |  |  |
|  | hsa04062:Chemokine signaling pathway | 2 | ***AM_HCC3_***(***AM^O^_CHB36-HCC3_***) | Yes (6) | / | Yes (6) |
|  |  |  | ***AM_CHB7_***(***AM^O^_CHB7-HCC20_***) |  |  |  |
|  | hsa04650:Natural killer cell mediated cytotoxicity | 2 | ***AM_HCC3_***(***AM^O^_CHB36-HCC3_***) | / | / | Yes (7) |
|  |  |  | ***AM_HCC3_***(***AM^O^_CHB7-HCC3_***) |  |  |  |
|  | hsa04620:Toll-like receptor signaling pathway | 1 | ***AM_CHB7_***(***AM^O^_CHB7-HCC3_***) | Yes (8) | / | Yes (9) |
|  | hsa04621:NOD-like receptor signaling pathway | 2 | ***AM_CHB7_***(***AM^O^_CHB7-HCC3_***) | not reported | / | Yes (10) |
|  |  |  | ***AM_CHB7_***(***AM^O^_CHB7-HCC20_***) |  |  |  |
|  | hsa04660:T cell receptor signaling pathway | 2 | ***AM_CHB7_***(***AM^O^_CHB7-HCC3_***) | Yes (11) | / | Yes (12) |
|  |  |  | ***AM_CHB7_***(***AM^O^_CHB7-HCC20_***) |  |  |  |
|  | hsa04612:Antigen processing and presentation | 2 | ***AM_CHB35_***(***AM^O^_CHB35-C13-HCC24_***) | Yes (13) | / | Yes (14) |
|  |  |  | ***AM_HCC24_***(***AM^O^_CHB35-C13-HCC24_***) |  |  |  |
| **Endocrine system** | hsa04914:Progesterone-mediated oocyte maturation | 3 | ***AM_C2_***(***AM^O^_C2-HCC20_***) | not reported | not reported | Yes (15) |
|  |  |  | ***AM_CHB7_***(***AM^O^_CHB7-HCC3_***) |  |  |  |
|  |  |  | ***AM_CHB7_***(***AM^O^_CHB7-HCC20_***) |  |  |  |
|  | hsa04910:Insulin signaling pathway | 3 | ***AM_C2_***(***AM^O^_C2-HCC20_***) | Yes (16) | not reported | not reported |
|  |  |  | ***AM_CHB7_***(***AM^O^_CHB7-HCC3_***) |  |  |  |
|  |  |  | ***AM_CHB7_***(***AM^O^_CHB7-HCC20_***) |  |  |  |
| **Signal transduction** | hsa04012:ErbB signaling pathway | 3 | ***AM_C2_***(***AM^O^_C2-HCC20_***) | Yes (17) | not reported | Yes (18) |
|  |  |  | ***AM_CHB7_***(***AM^O^_CHB7-HCC3_***) |  |  |  |
|  |  |  | ***AM_CHB7_***(***AM^O^_CHB7-HCC20_***) |  |  |  |
|  | hsa04010:MAPK signaling pathway | 1 | ***AM_C2_***(***AM^O^_C2-HCC20_***) | / | not reported | Yes (19) |
|  | hsa04370:VEGF signaling pathway | 2 | ***AM_HCC3_***(***AM^O^_CHB36-HCC3_***) | Yes (20) | / | Yes (19) |
|  |  |  | ***AM_CHB7_***(***AM^O^_CHB7-HCC20_***) |  |  |  |
|  | hsa04150:mTOR signaling pathway | 2 | ***AM_CHB7_***(***AM^O^_CHB7-HCC3_***) | Yes (20) | / | Yes (21) |
|  |  |  | ***AM_CHB7_***(***AM^O^_CHB7-HCC20_***) |  |  |  |
| **Nervous system** | hsa04722:Neurotrophin signaling pathway | 4 | ***AM_C2_***(***AM^O^_C2-HCC20_***) | Yes (22) | not reported | Yes (23) |
|  |  |  | ***AM_CHB7_***(***AM^O^_CHB7-HCC3_***) |  |  |  |
|  |  |  | ***AM_CHB7_***(***AM^O^_CHB7-HCC20_***) |  |  |  |
|  |  |  | ***AM_HCC12_***(***AM^O^_CHB9-HCC12_***) |  |  |  |
| **Membrane transport** | hsa02010:ABC transporters | 2 | ***AM_HCC20_***(***AM^O^_C2-HCC20_***) | / | / | Yes (24) |
|  |  |  | ***AM_HCC20_***(***AM^O^_CHB7-HCC20_***) |  |  |  |
| **Signaling molecules and interaction** | hsa04060:Cytokine-cytokine receptor interaction | 2 | ***AM_HCC20_***(***AM^O^_C2-HCC20_***) | / | / | Yes (25) |
|  |  |  | ***AM_HCC20_***(***AM^O^_CHB7-HCC20_***) |  |  |  |
| **Cell motility** | hsa04810:Regulation of actin cytoskeleton | 2 | ***AM_HCC3_***(***AM^O^_CHB36-HCC3_***) | / | / | Yes (26) |
|  |  |  | ***AM_HCC20_***(***AM^O^_CHB7-HCC20_***) |  |  |  |
| **Human Diseases; Cancers** | hsa05200:Pathways in cancer | 1 | ***AM_CHB7_***(***AM^O^_CHB7-HCC3_***) | Yes (27) | / | Yes (27) |
| **Cell growth and death** | hsa04210:Apoptosis | 1 | ***AM_CHB9_***(***AM^O^_CHB9-HCC12_***) | Yes (28) | / | Yes (29) |
|  |  |  |  |  |  |  |
|  |  |  |  |  |  |  |
|  |  |  |  |  |  |  |
| **Internal overlapping pathways** | | | | | | |
| **Metabolism** | hsa00982:Drug metabolism | 1 | ***AM_CHB11_*, *AM_HCC6_* (*AM^O^_CHB11-HCC6_*)** | not reported | / | not reported |
|  | hsa00591:Linoleic acid metabolism | 1 |  | Yes (30) | / | Yes (31) |
|  | hsa00830:Retinol metabolism | 1 |  | not reported | / | Yes (32) |
|  | hsa00980:Metabolism of xenobiotics by cytochrome P450 | 1 |  | Yes (33) | / | Yes (34) |
|  | hsa00590:Arachidonic acid metabolism | 1 |  | Yes (30) | / | Yes (31) |
|  | hsa00232:Caffeine metabolism | 1 |  | not reported | / | Yes (35) |
| **Immune system** | hsa04622:RIG-I-like receptor signaling pathway | 1 | ***AM_CHB53_*, *AM_HCC30_* (*AM^O^_CHB53-HCC30_*)** | Yes (36) | / | not reported |

**References**

1. Bouchard MJ, Wang L, Schneider RJ. Activation of focal adhesion kinase by hepatitis B virus HBx protein: multiple functions in viral replication. J Virol. 2006; 80:4406-14.

2. Fujii T, Koshikawa K, Nomoto S, Okochi O, Kaneko T, Inoue S, Yatabe Y, Takeda S, Nakao A. Focal adhesion kinase is overexpressed in hepatocellular carcinoma and can be served as an independent prognostic factor. J Hepatol. 2004; 41:104-11.

3. Lara-Pezzi E, Roche S, Andrisani OM, Sánchez-Madrid F, López-Cabrera M. The hepatitis B virus HBx protein induces adherens junction disruption in a src-dependent manner. Oncogene. 2001; 20:3323-31.

4. Cao Y, Chang H, Li L, Cheng RC, Fan XN. Alteration of adhesion molecule expression and cellular polarity in hepatocellular carcinoma. Histopathology. 2007; 51:528-38.

5. Lavi O, Skinner J, Gottesman MM. Network features suggest new hepatocellular carcinoma treatment strategies. BMC Syst Biol. 2014; 8:88.

6. Marra F, Tacke F. Roles for chemokines in liver disease. Gastroenterology. 2014; 147:577-94.

7. Tian Z, Chen Y, Gao B. Natural killer cells in liver disease. Hepatology. 2013; 57:1654-62.

8. Isogawa M, Robek MD, Furuichi Y, Chisari FV. Toll-like receptor signaling inhibits hepatitis B virus replication in vivo. J Virol. 2005; 79:7269-72.

9. Soares JB, Pimentel-Nunes P, Roncon-Albuquerque R, Leite-Moreira A. The role of lipopolysaccharide/toll-like receptor 4 signaling in chronic liver diseases. Hepatol Int. 2010; 4:659-72.

10. Saxena M, Yeretssian G. NOD-Like Receptors: Master Regulators of Inflammation and Cancer. Front Immunol. 2014; 5:327.

11. [Ye B](https://www.ncbi.nlm.nih.gov/pubmed/?term=Ye%20B%5BAuthor%5D&cauthor=true&cauthor_uid=25789969), [Liu X](https://www.ncbi.nlm.nih.gov/pubmed/?term=Liu%20X%5BAuthor%5D&cauthor=true&cauthor_uid=25789969), [Li X](https://www.ncbi.nlm.nih.gov/pubmed/?term=Li%20X%5BAuthor%5D&cauthor=true&cauthor_uid=25789969), [Kong H](https://www.ncbi.nlm.nih.gov/pubmed/?term=Kong%20H%5BAuthor%5D&cauthor=true&cauthor_uid=25789969), [Tian L](https://www.ncbi.nlm.nih.gov/pubmed/?term=Tian%20L%5BAuthor%5D&cauthor=true&cauthor_uid=25789969), [Chen Y](https://www.ncbi.nlm.nih.gov/pubmed/?term=Chen%20Y%5BAuthor%5D&cauthor=true&cauthor_uid=25789969). T-cell exhaustion in chronic hepatitis B infection: current knowledge and clinical significance. Cell Death Dis. 2015; 6:e1694.

12. Gehring AJ1, Ho ZZ, Tan AT, Aung MO, Lee KH, Tan KC, Lim SG, Bertoletti A. Profile of tumor antigen-specific CD8 T cells in patients with hepatitis B virus-related hepatocellular carcinoma. Gastroenterology. 2009; 137:682-90.

13. Sukriti S, Pati NT, Bose S, Hissar SS, Sarin SK. Impaired antigen processing and presentation machinery is associated with immunotolerant state in chronic hepatitis B virus infection. J Clin Immunol. 2010; 30:419-25.

14. Clifford RJ, Zhang J, Meerzaman DM, Lyu MS, Hu Y, Cultraro CM, Finney RP, Kelley JM, Efroni S, Greenblum SI, et al. Genetic variations at loci involved in the immune response are risk factors for hepatocellular carcinoma. Hepatology. 2010; 52:2034-43.

15. Wong YH, Wu CC, Lin CL, Chen TS, Chang TH, Chen BS. Applying NGS Data to Find Evolutionary Network Biomarkers from the Early and Late Stages of Hepatocellular Carcinoma. Biomed Res Int. 2015; 2015:391475.

16. Kim K, Kim KH, Cheong J. Hepatitis B virus X protein impairs hepatic insulin signaling through degradation of IRS1 and induction of SOCS3. PLoS One. 2010; 5:e8649.

17. Liu J, Ahiekpor A, Li L, Li X, Arbuthnot P, Kew M, Feitelson MA. Increased expression of ErbB-2 in liver is associated with hepatitis B x antigen and shorter survival in patients with liver cancer. Int J Cancer. 2009; 125:1894-901.

18. Arzumanyan A, Reis HM, Feitelson MA. Pathogenic mechanisms in HBV- and HCV-associated hepatocellular carcinoma. Nat Rev Cancer. 2013; 13:123-35.

19. Whittaker S, Marais R, Zhu AX. The role of signaling pathways in the development and treatment of hepatocellular carcinoma. Oncogene. 2010; 29:4989-5005.

20. Yen CJ, Lin YJ, Yen CS, Tsai HW, Tsai TF, Chang KY, Huang WC, Lin PW, Chiang CW, Chang TT. Hepatitis B virus X protein upregulates mTOR signaling through IKKβ to increase cell proliferation and VEGF production in hepatocellular carcinoma. PLoS One. 2012;7:e41931.

21. Villanueva A, Chiang DY, Newell P, Peix J, Thung S, Alsinet C, Tovar V, Roayaie S, Minguez B, Sole M, et al. Pivotal role of mTOR signaling in hepatocellular carcinoma. Gastroenterology. 2008; 135:1972-83, 1983.e1-11.

22. Amoras Eda S, Gomes ST, Freitas FB, Santana BB, Ishak G, de Araújo MT, Demachki S, da Silva Conde SR, de Oliveira Guimarães Ishak M, Ishak R, Vallinoto AC. NGF and P75NTR gene expression is associated with the hepatic fibrosis stage due to viral and non-viral causes. PLoS One. 2015; 10:e0121754.

23. Malaguarnera G, Giordano M, Paladina I, Berretta M, Cappellani A, Malaguarnera M. Serum markers of hepatocellular carcinoma. Dig Dis Sci. 2010; 55:2744-55.

24. Zen Y, Fujii T, Yoshikawa S, Takamura H, Tani T, Ohta T, Nakanuma Y. Histological and culture studies with respect to ABCG2 expression support the existence of a cancer cell hierarchy in human hepatocellular carcinoma. Am J Pathol. 2007; 170:1750-62.

25. Mosca E, Alfieri R, Milanesi L. Diffusion of Information throughout the Host Interactome Reveals Gene Expression Variations in Network Proximity to Target Proteins of Hepatitis C Virus. PLoS ONE. 2014; 9:e113660.

26. Yamaguchi H, Condeelis J. Regulation of the actin cytoskeleton in cancer cell migration and invasion. Biochim Biophys Acta. 2007; 1773:642-52.

27. Chen L, Liu R, Liu ZP, Li M, Aihara K. Detecting early-warning signals for sudden deterioration of complex diseases by dynamical network biomarkers. Sci Rep. 2012; 2: 342.

28. Miao J, Chen GG, Chun SY, Lai PP. Hepatitis B virus X protein induces apoptosis in hepatoma cells through inhibiting Bcl-xL expression. Cancer Lett. 2006; 236: 115-24.

29. Ikeguchi M, Hirooka Y, Kaibara N. Quantitative analysis of apoptosis-related gene expression in hepatocellular carcinoma. Cancer. 2002; 95: 1938-45.

30. Arain SQ, Talpur FN, Channa NA, Ali MS, Afridi HI. Serum lipids as an indicator for the alteration of liver function in patients with hepatitis B. Lipids Health Dis. 2018; 17:36.

31. Beyoğlu D, Imbeaud S, Maurhofer O, Bioulac-Sage P, Zucman-Rossi J, Dufour JF, Idle JR. Tissue metabolomics of hepatocellular carcinoma: tumor energy metabolism and the role of transcriptomic classification. Hepatology. 2013; 58:229-38.

32. Yuan JM, Gao YT, Ong CN, Ross RK, Yu MC. Prediagnostic level of serum retinol in relation to reduced risk of hepatocellular carcinoma. J Natl Cancer Inst. 2006; 98: 482-90.

33. Liu H, Lou G, Li C, Wang X, Cederbaum AI, Gan L, Xie B. HBx inhibits CYP2E1 gene expression via downregulating HNF4α in human hepatoma cells. PLoS One. 2014; 9:e107913.

34. Kinoshita M, Miyata M. Underexpression of mRNA in human hepatocellular carcinoma focusing on eight loci. Hepatology. 2002; 36:433-8.

35. Muriel P, Arauz J. Coffee and liver diseases. Fitoterapia. 2010; 81: 297-305.

36. Wang H, Ryu WS. Hepatitis B virus polymerase blocks pattern recognition receptor signaling via interaction with DDX3: implications for immune evasion. PLoS Pathog. 2010; 6:e1000986.

**Table S5 Highly correlated gene pairs and top 5** **important genes in the 13 *OAMs***

| ***OAMs*** | **OOB classification error rates for predicting CHB** | **OOB classification error rates for predicting cirrhosis** | **OOB classification error rates for predicting HCC** | **At least one OOB classification error rate < 0.5** | **The frequency of** **highly correlated gene pairs (r>0.8, P-value=0) appearing in the *OAMs*** | **Both top 5 important genes and highly correlated gene pairs in the *OAMs*** |
| --- | --- | --- | --- | --- | --- | --- |
| ***AM^O^_C16-HCC35_*** | 0.5 | 0.38 | 0.8 | √ | 0 | * |
| ***AM^O^_CHB5-C3-HCC10_*** | 0.6 | 0.38 | 0.8 | √ | 0 | * |
| ***AM^O^_CHB36-HCC3_*** | 0.4 | 0.38 | 0.9 | √ | 4 | decr1-pik3ca; decr1-rac1; pik3ca-rac1 |
| ***AM^O^_CHB11-HCC6_*** | 0.7 | 0.46 | 0.7 | √ | 5 | cyp2c9-cyp2c19; cyp2c9-cyp1a2 |
| ***AM^O^_CHB53-HCC30_*** | 0.4 | 0.62 | 1.0 | √ | 0 | * |
| ***AM^O^_C2-HCC20_*** | 0.5 | 0.23 | 0.9 | √ | 4 | pik3ca-mapk8; mapk8-il6 |
| ***AM^O^_C21-HCC57_*** | 0.5 | 0.54 | 0.9 | × | 2 | ** |
| ***AM^O^_CHB14-HCC21_*** | 0.6 | 0.31 | 0.9 | √ | 1 | mgmt-socs1 |
| ***AM^O^_CHB9-HCC12_*** | 0.3 | 0.31 | 0.6 | √ | 4 | diablo-ebp; tnfrsf10b-ebp |
| ***AM^O^_CHB7-HCC3_*** | 0.6 | 0.38 | 1.0 | √ | 6 | pik3ca-rac1 |
| ***AM^O^_CHB7-HCC20_*** | 0.5 | 0.38 | 0.9 | √ | 4 | ** |
| ***AM^O^_CHB23-C11-HCC38_*** | 0.5 | 0.46 | 1.0 | √ | 9 | hdac2-prkaa1 |
| ***AM^O^_CHB35-C13-HCC24_*** | 0.4 | 0.31 | 0.9 | √ | 3 | ** |

* No highly correlated gene pairs were included in the *OAMs.*

** The highly correlated gene pairs in the *OAMs* were not the top 5 important genes in the *OAMs.*

**Table S5-1 The 39 highly correlated gene pairs(r>0.8, P-value=0) appeared in 10 *OAMs***

|  | **Gene pairs** | **Lower quartile** | **Correlation coefficient** | **Upper quartile** | **P-value** | **The *OAMs* which highly correlated gene pairs appeared in** |
| --- | --- | --- | --- | --- | --- | --- |
| 1 | tgf11-mmp2 | 0.966 | 0.983 | 0.992 | 0 | *AM^O^_CHB7-HCC3_* |
| 2 | klrd1-klrk1 | 0.913 | 0.956 | 0.978 | 0 | *AM^O^_CHB35-C13-HCC24_* |
| 3 | tfam-hdac2 | 0.853 | 0.925 | 0.963 | 0 | *AM^O^_CHB23-C11-HCC38_* |
| 4 | hdac2-prka1 | 0.853 | 0.925 | 0.963 | 0 | *AM^O^_CHB23-C11-HCC38_* |
| 5 | uts2r-saa | 0.845 | 0.921 | 0.961 | 0 | *AM^O^_C2-HCC20_* |
| 6 | tgf11-ccl2 | 0.841 | 0.919 | 0.959 | 0 | *AM^O^_CHB7-HCC3_* |
| 7 | cyp29-cyp2b6 | 0.834 | 0.915 | 0.958 | 0 | *AM^O^_CHB11-HCC6_* |
| 8 | cyp29-cyp12 | 0.83 | 0.913 | 0.957 | 0 | *AM^O^_CHB11-HCC6_* |
| 9 | ccl2-mmp2 | 0.83 | 0.913 | 0.957 | 0 | *AM^O^_CHB7-HCC3_; AM^O^_CHB7-HCC20_* |
| 10 | ptk2-il6 | 0.828 | 0.912 | 0.956 | 0 | *AM^O^_CHB7-HCC20_* |
| 11 | lyn-syk | 0.824 | 0.91 | 0.955 | 0 | *AM^O^_CHB36-HCC3_; AM^O^_CHB7-HCC3_* |
| 12 | mmp2-timp2 | 0.824 | 0.91 | 0.955 | 0 | *AM^O^_CHB23-C11-HCC38_* |
| 13 | mgmt-socs1 | 0.797 | 0.895 | 0.947 | 0 | *AM^O^_CHB14-HCC21_* |
| 14 | il6-ccl2 | 0.795 | 0.894 | 0.947 | 0 | *AM^O^_CHB7-HCC20_* |
| 15 | pparg-timp2 | 0.784 | 0.888 | 0.944 | 0 | *AM^O^_CHB23-C11-HCC38_* |
| 16 | klrd1-klrc1 | 0.778 | 0.885 | 0.942 | 0 | *AM^O^_CHB35-C13-HCC24_* |
| 17 | tnfr10-atf6 | 0.77 | 0.88 | 0.94 | 0 | *AM^O^_CHB9-HCC12_* |
| 18 | krt18-tfam | 0.767 | 0.879 | 0.939 | 0 | *AM^O^_CHB23-C11-HCC38_* |
| 19 | parp1-foxm1 | 0.745 | 0.867 | 0.932 | 0 | *AM^O^_C21-HCC57_* |
| 20 | cyp29-cyp28 | 0.741 | 0.864 | 0.931 | 0 | *AM^O^_CHB11-HCC6_* |
| 21 | tnfr10-ebp | 0.738 | 0.863 | 0.931 | 0 | *AM^O^_CHB9-HCC12_* |
| 22 | diabl-ebp | 0.719 | 0.852 | 0.925 | 0 | *AM^O^_CHB9-HCC12_* |
| 23 | tfam-prka1 | 0.718 | 0.852 | 0.925 | 0 | *AM^O^_CHB23-C11-HCC38_* |
| 24 | gna12-mica | 0.712 | 0.848 | 0.923 | 0 | *AM^O^_CHB35-C13-HCC24_* |
| 25 | decr1-pik3c | 0.709 | 0.846 | 0.922 | 0 | *AM^O^_CHB36-HCC3_* |
| 26 | mapk8-il6 | 0.705 | 0.844 | 0.921 | 0 | *AM^O^_C2-HCC20_* |
| 27 | birc3-casp8 | 0.696 | 0.839 | 0.918 | 0 | *AM^O^_C21-HCC57_* |
| 28 | krt18-hdac2 | 0.695 | 0.838 | 0.917 | 0 | *AM^O^_CHB23-C11-HCC38_* |
| 29 | mapk8-tnfr10 | 0.693 | 0.837 | 0.917 | 0 | *AM^O^_CHB9-HCC12_* |
| 30 | sgsm3-abcb6 | 0.681 | 0.83 | 0.913 | 0 | *AM^O^_C2-HCC20_* |
| 31 | decr1-rac1 | 0.676 | 0.827 | 0.912 | 0 | *AM^O^_CHB36-HCC3_* |
| 32 | pik3c-rac1 | 0.67 | 0.824 | 0.91 | 0 | *AM^O^_CHB36-HCC3_; AM^O^_CHB7-HCC3_* |
| 33 | cyp29-cy219 | 0.67 | 0.824 | 0.91 | 0 | *AM^O^_CHB11-HCC6_* |
| 34 | pik3c-mapk8 | 0.652 | 0.813 | 0.904 | 0 | *AM^O^_C2-HCC20_* |
| 35 | lyn-abcb1 | 0.65 | 0.812 | 0.904 | 0 | *AM^O^_CHB7-HCC20_* |
| 36 | rac1-ccl2 | 0.649 | 0.812 | 0.903 | 0 | *AM^O^_CHB7-HCC3_* |
| 37 | cyp2a6-cyp2b6 | 0.632 | 0.801 | 0.898 | 0 | *AM^O^_CHB11-HCC6_* |
| 38 | mmp2-pparg | 0.632 | 0.801 | 0.898 | 0 | *AM^O^_CHB23-C11-HCC38_* |
| 39 | krt16-hdac3 | -0.905 | -0.814 | -0.654 | 0 | *AM^O^_CHB23-C11-HCC38_* |

**Table S6** **The associations between highly correlated genes in 13 *OAMs* and three diseases**

| ***OAMs*** | **Genes** | **Also known as** | **Chronic hepatitis B** | **Cirrhosis** | **Hepatocellular carcinoma** |
| --- | --- | --- | --- | --- | --- |
| ***AM^O^_CHB36-HCC3_*** | **decr1** | **-** | not reported | - | These include seminoma, hepatocellular carcinoma, clear renal cell carcinoma, pancreatic adenocarcinoma, ovarian adenocarcinoma, and colon carcinoma, all of which exhibit dramatically reduced levels in DecR1 expression relative to their normal tissue counterparts(1). |
| ***AM^O^_CHB36-HCC3_***, ***AM^O^_C2-HCC20_*** | **pik3ca** | **PI3K** | Indeed, HBx was reported previously to activate the PI3K-Akt pathway. (2) HBc expression activates the Src/PI3K/Akt signaling pathway in hepatoma cells. | Suppression of PI3K/AKT/mTOR/p70S6K1 signaling pathways could be an important strategy in the prevention and treatment of liver fibrosis (3). | Controversial reports have been published on the presence of somatic mutations in the exon 9 of PIK3CA gene (4).  The PIK3CA rs17849071 and rs17849079 polymorphisms may increase the risk of HCC either independently or synergistically (5). |
| ***AM^O^_CHB36-HCC3_***, ***AM^O^_CHB7-HCC3_*** | **rac1** | **-** | Rac1 GTPase is activated by hepatitis B virus replication — involvement of HBX (6). | - | Rac1, which has been shown to be involved in cancer cell metastasis, is highly expressed in aggressive HCC cell lines and its activity correlated with cell motility and cytoskeleton polymerization (7). |
| ***AM^O^_CHB11-HCC6_*** | **cyp2c9** | **-** | The expressions of CYP2C9 mRNA and protein are decreased in chronic HBV infection which may down-regulate the enzyme activity (8). | - | CYP2C9, one of the most abundant and important DMEs, is involved in the metabolism of many carcinogens and drugs and is down-regulated in HCC (9). |
| ***AM^O^_CHB11-HCC6_*** | **cyp2c19** | **-** | The expressions of 12 other CYPs, including CYP2A6, CYP2A7 and CYP2C19, were down-regulated in HBV- and/or HCV-infected livers compared with normal livers (10). | - | It has also been shown that the genetic polymorphisms of CYP2E, CYP2D6 and CYP2C19 are associated with the development of HCC (10). |
| ***AM^O^_CHB11-HCC6_*** | **cyp1a2** | **-** | The CYP1A2 activity significantly decreased in the patients with cirrhosis or chronic hepatitis B (11). | - | CYP1A2 genetic polymorphisms are associated with HCC susceptibility in smokers and HBsAg seronegative individuals in the Fusui endemic region (12). |
| ***AM^O^_C2-HCC20_*** | **mapk8** | **JNK1** | - | JNK1 may be associated with the induction of diet-induced steatohepatitis and liver fibrosis (13). | JNK1, a potential therapeutic target for hepatocellular carcinoma. Recently, several groups have attributed an important role for c-Jun N-terminal kinase 1 (JNK1) in the pathogenesis of human HCC and its close association with the expression of HCC signature genes (14). |
| ***AM^O^_C2-HCC20_*** | **il6** | **-** | - | Serum ghrelin, TNF-α, and IL-6 levels were significantly higher in cirrhosis and HCC groups compared to in the control group (P < .05) (15). | In conclusion, the IL-6 levels in patients with LC-associated HCC are higher than in patients with LC alone and controls, indicating that production of this cytokine is increased by tumor cells (16). |
| ***AM^O^_CHB14-HCC21_*** | **mgmt** | **-** | not reported | - | These results suggest that the risk of HCC is related to P14 methylation, but not MGMT methylation (17). |
| ***AM^O^_CHB14-HCC21_*** | **socs1** | **-** | Our results suggested that the gene loss and epigenetic silencing of SOCS1 were strongly associated with HBV-related HCC (18). | - | RASSF1A and SOCS1 genes methylation status may play an important role in the process of hepatocarcinogenesis and may be used as diagnostic and prognostic noninvasive biomarkers for HCC when combined with serum AFP (19). |
| ***AM^O^_CHB9-HCC12_*** | **diablo** | **-** | not reported | - | Characteristic genes expressed in CH-B-related HCC: Diablo, IAP-binding mitochondrial protein DIABLO NM_019887 13, Down-regulated (20). |
| ***AM^O^_CHB9-HCC12_*** | **ebp** | **-** | not reported | - | not reported |
| ***AM^O^_CHB9-HCC12_*** | **tnfrsf10b** | **DR5,** **TRAILR2** | We demonstrate that the expression of the TNFRSF10B protein is dramatically reduced both in liver tissues of chronic hepatitis B patients and in cell lines transfected with HBV or HBx (21). | - | The expression of TRAIL-R1 and TRAIL-R2 was down-regulated in HCCs tissues compared with the adjacent non-malignant liver (22). |
| ***AM^O^_CHB23-C11-HCC38_*** | **hdac2** | - | not reported | not reported | Taken together, our study suggests that both HDAC1 and HDAC2 exert pro-survival effects in HCC cells. HDAC1 and HDAC2 independently predict mortality in HCC (23). |
| ***AM^O^_CHB23-C11-HCC38_*** | **prkaa1** | **AMPK; AMPKa1** | These findings suggest that PRKAA1 polymorphisms may contribute to the susceptibility of chronic HBV infection in Chinese Han origin (24). | AMPK: a novel target for treating hepatic fibrosis (25). AMPK activation seems to be a potential target for the prevention of hepatocellular carcinoma in patients with liver cirrhosis (26). | AMPK is dysfunctional in patients with HCC, and low p-AMPK staining is correlated with aggressive clinicopathologic features and poor prognosis (27). |

**References**

1. Ursini-Siegel J, Rajput AB, Lu H, Sanguin-Gendreau V, Zuo D, Papavasiliou V, Lavoie C, Turpin J, Cianflone K, Huntsman DG, Muller WJ. Elevated expression of DecR1 impairs ErbB2/Neu-induced mammary tumor development. Mol Cell Biol. 2007; 27: 6361-71.

2. Liu W, Guo TF, Jing ZT, Yang Z, Liu L, Yang YP, Lin X, Tong QY. Hepatitis B virus core protein promotes hepatocarcinogenesis by enhancing Src expression and activating the Src/PI3K/Akt pathway. FASEB J. 2018; 32:3033-46.

3. Peng R, Wang S, Wang R, Wang Y, Wu Y, Yuan Y. Antifibrotic effects of tanshinol in experimental hepatic fibrosis by targeting PI3K/AKT/mTOR/p70S6K1 signaling pathways. Discov Med. 2017; 23: 81-94.

4. Tornesello ML, Buonaguro L, Tatangelo F, Botti G, Izzo F, Buonaguro FM. Mutations in TP53, CTNNB1 and PIK3CA genes in hepatocellular carcinoma associated with hepatitis B and hepatitis C virus infections. Genomics. 2013; 102:74-83.

5. Li HG, Liu FF, Zhu HQ, Zhou X, Lu J, Chang H, Hu JH. PIK3CA polymorphisms associated with susceptibility to hepatocellular carcinoma. Int J Clin Exp Pathol. 2015; 8:15255-9.

6. Tan TL, Fang N, Neo TL, Singh P, Zhang J, Zhou R, Koh CG, Chan V, Lim SG, Chen WN. Rac1 GTPase is activated by hepatitis B virus replication--involvement of HBX. Biochim Biophys Acta. 2008; 1783:360-74.

7. Cao X, Zhang L, Shi Y, Sun Y, Dai S, Guo C, Zhu F, Wang Q, Wang J, Wang X, et al. Human tumor necrosis factor (TNF)-alpha-induced protein 8-like 2 suppresses hepatocellular carcinoma metastasis through inhibiting Rac1. Mol Cancer. 2013; 12:149.

8. Zhou F, Miao X, Gong Z, Yao J, Wu NI, Hu Z. Effects of chronic hepatitis B virus infection on human hepatic cytochrome P450 2C9. Chin J Infect Dis. 2009; 27:94-98.

9. Yu D, Green B, Marrone A, Guo Y, Kadlubar S, Lin D, Fuscoe J, Pogribny I, Ning B. Suppression of CYP2C9 by microRNA hsa-miR-128-3p in human liver cells and association with hepatocellular carcinoma. Sci Rep. 2015; 5:8534.

10. Iizuka N, Oka M, Hamamoto Y, Mori N, Tamesa T, Tangoku A, Miyamoto T, Uchimura S, Nakayama H, Hamada K, Yamada-Okabe H. Altered Levels of Cytochrome P450 Genes in Hepatitis B or C Virus-infected Liver Identified by Oligonucleotide Microarray. Cancer Genomics Proteomics. 2004; 1:53-8.

11. Wang XR, Qu ZQ, Li XD, Liu HL, He P, Fang BX, Xiao J, Huang W, Wu MC. Activity of sulfotransferase 1A1 is dramatically upregulated in patients with hepatocellular carcinoma secondary to chronic hepatitis B virus infection. Cancer Sci. 2010; 101: 412-5.

12. Chen X, Wang H, Xie W, Liang R, Wei Z, Zhi L, Zhang X, Hao B, Zhong S, Zhou G, et al. Association of CYP1A2 genetic polymorphisms with hepatocellular carcinoma susceptibility: a case-control study in a high-risk region of China. Pharmacogenet Genomics. 2006; 16:219-27.

13. Maeda S. NF-κB, JNK, and TLR Signaling Pathways in Hepatocarcinogenesis. Gastroenterol Res Pract. 2010; 2010:367694.

14. Chen F, Beezhold K, Castranova V. JNK1, a potential therapeutic target for hepatocellular carcinoma. Biochim Biophys Acta. 2009; 1796:242-51.

15. Ataseven H, Bahcecioglu IH, Kuzu N, Yalniz M, Celebi S, Erensoy A, Ustundag B. The levels of ghrelin, leptin, TNF-alpha, and IL-6 in liver cirrhosis and hepatocellular carcinoma due to HBV and HDV infection. Mediators Inflamm. 2006; 2006:78380.

16. Soresi M, Giannitrapani L, D'Antona F, Florena AM, La Spada E, Terranova A, Cervello M, D'Alessandro N, Montalto G. Interleukin-6 and its soluble receptor in patients with liver cirrhosis and hepatocellular carcinoma. World J Gastroenterol. 2006; 12:2563-8.

17. Li CC, Yu Z, Cui LH, Piao JM, Liu M. Role of P14 and MGMT gene methylation in hepatocellular carcinomas: a meta-analysis. Asian Pac J Cancer Prev. 2014;15:6591-6.

18. Zhang X, Wang J, Cheng J, Ding S, Li M, Sun S, Zhang L, Liu S, Chen X, Zhuang H, Lu F. An integrated analysis of SOCS1 down-regulation in HBV infection-related hepatocellular carcinoma. J Viral Hepat. 2014; 21:264-71.

19. Pasha HF, Mohamed RH, Radwan MI. RASSF1A and SOCS1 genes methylation status as a noninvasive marker for hepatocellular carcinoma. Cancer Biomark. 2019; 24: 241-247.

20. Ueda T, Honda M, Horimoto K, Aburatani S, Saito S, Yamashita T, Sakai Y, Nakamura M, Takatori H, Sunagozaka H, Kaneko S. Gene expression profiling of hepatitis B- and hepatitis C-related hepatocellular carcinoma using graphical Gaussian modeling. Genomics. 2013; 101: 238-48.

21. Shin GC, Kang HS, Lee AR1, Kim KH. Hepatitis B virus-triggered autophagy targets TNFRSF10B/death receptor 5 for degradation to limit TNFSF10/TRAIL response. Autophagy. 2016; 12:2451-66.

22. Shin EC, Seong YR, Kim CH, Kim H, Ahn YS, Kim K, Kim SJ, Hong SS, Park JH. Human hepatocellular carcinoma cells resist to TRAIL-induced apoptosis, and the resistance is abolished by cisplatin. Exp Mol Med. 2002; 34: 114-22.

23. Ler SY, Leung CH, Khin LW, Lu GD, Salto-Tellez M, Hartman M, Iau PT, Yap CT, Hooi SC. HDAC1 and HDAC2 independently predict mortality in hepatocellular carcinoma by a competing risk regression model in a Southeast Asian population. Oncol Rep. 2015; 34:2238-50.

24. Yuan J, Zhang Y, Yan FT, Zheng X. Association of PRKAA1 gene polymorphisms with chronic hepatitis B virus infection in Chinese Han population. Braz J Infect Dis. 2016; 20: 564-8.

25. Liang Z, Li T, Jiang S, Xu J, Di W, Yang Z, Hu W, Yang Y. AMPK: a novel target for treating hepatic fibrosis. Oncotarget. 2017; 8: 62780-92.

26. Yang X, Liu Y, Li M, Wu H, Wang Y, You Y, Li P, Ding X, Liu C, Gong J. Predictive and preventive significance of AMPK activation on hepatocarcinogenesis in patients with liver cirrhosis. Cell Death Dis. 2018; 9: 264.

27. Zheng L, Yang W, Wu F, Wang C, Yu L, Tang L, Qiu B, Li Y, Guo L, Wu M, et al. Prognostic significance of AMPK activation and therapeutic effects of metformin in hepatocellular carcinoma. Clin Cancer Res. 2013; 19: 5372-80.

**Table S7 The relationships between 15 genes and HCC biomarkers in literature**

| **Genes** | **Also known as** | **Relationships between the genes and HCC biomarkers in literature** |
| --- | --- | --- |
| **decr1** | - | not reported |
| **pik3ca** | PI3K | not reported |
| **rac1** |  | not reported |
| **cyp2c19** | - | Therefore, we identified CYP2C19 as a novel, potential clinically useful biomarker for the RFS of HCC [1]. Although these mechanisms should be verified in the future, we have confirmed the utility of CYP2C19 protein as a novel biomarker for predicting the prognosis in daily clinical practice [1]. |
| **cyp2c9** | - | Bioinformatic analysis reveals CYP2C9 as a potential prognostic marker for HCC and liver cancer cell lines suitable for its mechanism study [2]. |
| **cyp1a2** | - | Further prospective, multicenter study validated that noncancerous CYP1A2 expression was identified as a unique biomarker for the prediction of recurrence after the curative resection of early-stage HCC [3]. |
| **mapk8** | JNK; JNK1; | The status of JNK1 activation in HCC tissue, thus, might be a new biomarker for HCC prognosis and therapeutic targeting [4]. |
| **il6** | - | Therefore, IL-6 could be considered a HCC biomarker and blockade of IL-6 pathway may be a promising therapeutic alternative for HCC [5]. |
| **mgmt** | - | not reported |
| **socs1** | - | Our findings provide empirical evidence that abnormal SOCS-1 promoter methylation may contribute to the pathogenesis of HCC. Thus, detection of SOCS-1 promoter methylation may be a valuable diagnostic biomarker for HCC [6]. |
| **diablo** | - | The proteins listed in table 2 through table 7 show specific overexpression and underexpression in E1 and E2 HCC tissues compared to normal liver tissues and thus they can be used as proteomic marker for early detection of liver cancer.  DBLOH HUMAN (DIABLO protein; NCBI GI: 13325198) [7]; |
| **ebp** | - | not reported |
| **tnfrsf10b** | - | not reported |
| **hdac2** | - | HDAC2 is an independent predictor of survival in HCC. These differences in etiology could also explain our findings that HDAC2, but not HDAC1 or HDAC3, were prognostic markers in our cases [8]. |
| **prkaa1** | AMPK | In conclusion, the key contribution of our study is to identify p-AMPK as a valuable prognostic biomarker in predicting the outcome of HCC patients with TACE treatment [9]. |

**References**

1. Ashida R, Okamura Y, Ohshima K, Kakuda Y, Uesaka K, Sugiura T, Ito T, Yamamoto Y, Sugino T, Urakami K, Kusuhara M, Yamaguchi K. The down-regulation of the CYP2C19 gene is associated with aggressive tumor potential and the poorer recurrence-free survival of hepatocellular carcinoma. Oncotarget. 2018; 9: 22058-68.

2. Shuaichen L, Guangyi W. Bioinformatic analysis reveals CYP2C9 as a potential prognostic marker for HCC and liver cancer cell lines suitable for its mechanism study. Cell Mol Biol (Noisy-le-grand). 2018; 64:70-4.

3. Tanaka S, Mogushi K, Yasen M, Ban D, Noguchi N, Irie T, Kudo A, Nakamura N, Tanaka H, Yamamoto M, Kokudo N, Takayama T, Kawasaki S, Sakamoto M, Arii S. Oxidative stress pathways in noncancerous human liver tissue to predict hepatocellular carcinoma recurrence: a prospective, multicenter study. Hepatology. 2011; 54: 1273-81.

4. Chang Q, Chen J, Beezhold KJ, Castranova V, Shi X, Chen F. JNK1 activation predicts the prognostic outcome of the human hepatocellular carcinoma. Mol Cancer. 2009; 8:64.

5. Liu Y, Fuchs J, Li C, Lin J. IL-6, a risk factor for hepatocellular carcinoma: FLLL32 inhibits IL-6-induced STAT3 phosphorylation in human hepatocellular cancer cells. Cell Cycle. 2010; 9:3423-7.

6. Zhao RC, Zhou J, He JY, Wei YG, Qin Y, Li B. Aberrant promoter methylation of SOCS-1 gene may contribute to the pathogenesis of hepatocellular carcinoma: a meta-analysis. J BUON. 2016; 21:142-51.

7. Park J Y, Hong S J, Kim J M, Shim Y. Proteinic marker for early diagnosis of liver cancer: U.S. Patent 8,937,165[P]. 2015-1-20.

8. Quint K, Agaimy A, Di Fazio P, Montalbano R, Steindorf C, Jung R, Hellerbrand C, Hartmann A, Sitter H, Neureiter D, Ocker M. Clinical significance of histone deacetylases 1, 2, 3, and 7: HDAC2 is an independent predictor of survival in HCC. Virchows Arch. 2011; 459:129-39.

9. Zheng LY, Wu L, Lu J, Zou DJ, Huang Q. Expression of Phosphorylated AMP-Activated Protein Kinase Predicts Response to Transarterial Chemoembolization in Postoperative Cases of Hepatocellular Carcinoma. Medicine (Baltimore). 2016; 95:e2908.

**Table S8 The classification evaluation indexes of candidate genes and gene combinations for HCC identification**

| **Gene combinations** | **Total OOB error rates** | **AUC** | **G-mean** | **F-value** | **Sensitivity** | **Precision** | **Specificity** | **Accuracy** |
| --- | --- | --- | --- | --- | --- | --- | --- | --- |
| **rac1-il6-diablo** | 14.29% | 0.8210 | 0.2979 | 0.9318 | 0.9762 | 0.8913 | 0.0909 | 0.8737 |
| **cyp2c9-diablo-il6** | 11.25% | 0.9650 | 0.7253 | 0.9530 | 0.9643 | 0.9419 | 0.5455 | 0.9158 |
| **cyp2c9-diablo-rac1** | 9.12% | 0.9570 | 0.7342 | 0.9651 | 0.9881 | 0.9432 | 0.5455 | 0.9368 |
| **cyp2c19-diablo-il6** | 7.90% | 0.9250 | 0.7297 | 0.9591 | 0.9762 | 0.9425 | 0.5455 | 0.9263 |
| **cyp2c19-diablo-rac1** | 7.29% | 0.8810 | 0.6701 | 0.9595 | 0.9881 | 0.9326 | 0.4545 | 0.9263 |
| **cyp2c19-cyp2c9-diablo** | 7.90% | 0.9760 | 0.8426 | 0.9704 | 0.9762 | 0.9647 | 0.7273 | 0.9474 |
| **cyp1a2-diablo-il6** | 8.21% | 0.9730 | 0.8937 | 0.9762 | 0.9762 | 0.9762 | 0.8182 | 0.9579 |
| **cyp1a2-rac1-diablo** | 7.60% | 0.9620 | 0.7208 | 0.9468 | 0.9524 | 0.9412 | 0.5455 | 0.9053 |
| **cyp1a2-diablo-cyp2c9** | 7.29% | 0.9700 | 0.7785 | 0.9524 | 0.9524 | 0.9524 | 0.6364 | 0.9158 |
| **cyp1a2-cyp2c19-diablo** | 6.38% | 0.9840 | 0.8426 | 0.9704 | 0.9762 | 0.9647 | 0.7273 | 0.9474 |
| **cyp2c9-rac1-il6** | 10.03% | 0.9300 | 0.5096 | 0.9303 | 0.9524 | 0.9090 | 0.2727 | 0.8737 |
| **cyp2c19-rac1-il6** | 6.99% | 0.9210 | 0.6661 | 0.9535 | 0.9762 | 0.9318 | 0.4545 | 0.9158 |
| **cyp2c19-cyp2c9-il6** | 9.42% | 0.9530 | 0.8375 | 0.9643 | 0.9643 | 0.9643 | 0.7273 | 0.9368 |
| **cyp2c19-cyp2c9-rac1** | 8.51% | 0.9320 | 0.5885 | 0.9357 | 0.9524 | 0.9195 | 0.3636 | 0.8842 |
| **cyp1a2-rac1-il6** | 10.03% | 0.9710 | 0.7297 | 0.9591 | 0.9762 | 0.9425 | 0.5455 | 0.9263 |
| **cyp1a2-cyp2c9-il6** | 9.73% | 0.9720 | 0.8323 | 0.9581 | 0.9524 | 0.9638 | 0.7273 | 0.9263 |
| **cyp1a2-cyp2c9-rac1** | 9.73% | 0.9620 | 0.5885 | 0.9357 | 0.9524 | 0.9195 | 0.3636 | 0.8842 |
| **cyp1a2-cyp2c19-il6** | 5.78% | 0.9730 | 0.9305 | 0.9697 | 0.9524 | 0.9877 | 0.9091 | 0.9474 |
| **cyp1a2-cyp2c19-rac1** | 5.78% | 0.9700 | 0.7930 | 0.9707 | 0.9881 | 0.9540 | 0.6364 | 0.9474 |
| **cyp1a2-cyp2c19-cyp2c9** | 8.21% | 0.9740 | 0.7882 | 0.9647 | 0.9762 | 0.9535 | 0.6364 | 0.9368 |
| **diablo-il6** | 15.81% | 0.6530 | 0.2905 | 0.9070 | 0.9286 | 0.8864 | 0.0909 | 0.8316 |
| **rac1-diablo** | 13.37% | 0.7980 | 0.4082 | 0.9059 | 0.9167 | 0.8953 | 0.1818 | 0.8316 |
| **cyp2c9-diablo** | 10.94% | 0.9700 | 0.8937 | 0.9762 | 0.9762 | 0.9762 | 0.8182 | 0.9579 |
| **cyp2c19-diablo** | 7.90% | 0.8870 | 0.7785 | 0.9524 | 0.9524 | 0.9524 | 0.6364 | 0.9158 |
| **cyp1a2-diablo** | 7.60% | 0.9510 | 0.7687 | 0.9398 | 0.9286 | 0.9512 | 0.6364 | 0.8947 |
| **cyp1a2-cyp2c19** | 7.60% | 0.9630 | 0.7882 | 0.9647 | 0.9762 | 0.9535 | 0.6364 | 0.9368 |
| **cyp1a2-cyp2c9** | 10.64% | 0.9490 | 0.7785 | 0.9524 | 0.9524 | 0.9524 | 0.6364 | 0.9158 |
| **cyp1a2-rac1** | 8.21% | 0.9560 | 0.7208 | 0.9468 | 0.9524 | 0.9412 | 0.5455 | 0.9053 |
| **cyp1a2-il6** | 13.98% | 0.9640 | 0.7785 | 0.9524 | 0.9524 | 0.9524 | 0.6364 | 0.9158 |
| **cyp2c19-cyp2c9** | 10.94% | 0.9430 | 0.7834 | 0.9586 | 0.9643 | 0.9529 | 0.6364 | 0.9263 |
| **cyp2c19-rac1** | 9.12% | 0.7510 | 0.5921 | 0.9419 | 0.9643 | 0.9205 | 0.3636 | 0.8947 |
| **cyp2c19-il6** | 10.64% | 0.8720 | 0.7736 | 0.9461 | 0.9405 | 0.9518 | 0.6364 | 0.9053 |
| **cyp2c9-rac1** | 10.64% | 0.8890 | 0.5921 | 0.9419 | 0.9643 | 0.9205 | 0.3636 | 0.8947 |
| **cyp2c9-il6** | 13.98% | 0.9060 | 0.5032 | 0.9177 | 0.9286 | 0.9070 | 0.2727 | 0.8526 |
| **rac1-il6** | 14.59% | 0.7650 | 0.2979 | 0.9318 | 0.9762 | 0.8913 | 0.0909 | 0.8737 |
| **diablo** | 18.54% | 0.8200 | 0.5000 | 0.9113 | 0.9167 | 0.9059 | 0.2727 | 0.8421 |
| **cyp1a2** | 12.77% | 0.9440 | 0.6579 | 0.9412 | 0.9524 | 0.9302 | 0.4545 | 0.8947 |
| **cyp2c19** | 11.85% | 0.7060 | 0.5064 | 0.9240 | 0.9405 | 0.9080 | 0.2727 | 0.8632 |
| **cyp2c9** | 16.72% | 0.8830 | 0.5000 | 0.9113 | 0.9167 | 0.9059 | 0.2727 | 0.8421 |
| **rac1** | 16.41% | 0.6220 | 0.4967 | 0.9048 | 0.9048 | 0.9048 | 0.2727 | 0.8316 |
| **il6** | 16.41% | 0.6040 | 0.2979 | 0.9318 | 0.9762 | 0.8913 | 0.0909 | 0.8737 |

**Table S8-1 Descriptive statistics and intergroup comparisons summarizing the classification evaluation indexes**

| **Index** | **Two gene combinations (N=15)** | **Three gene combinations (N=20)** | **Total (N=35)** | **Group test** | **P value** |
| --- | --- | --- | --- | --- | --- |
| **train-OOB-error** |  |  |  | Kruskal-Wallis rank sum test | 0.004 |
| Mean (SD) | 0.11 (0.03) | 0.08 (0.02) | 0.10 (0.03) |  |  |
| Median (Q1, Q3) | 0.11 (0.09, 0.14) | 0.08 (0.07, 0.09) | 0.09 (0.08, 0.11) |  |  |
| Min - Max | 0.08 - 0.16 | 0.06 - 0.14 | 0.06 - 0.16 |  |  |
| **AUC** |  |  |  | Kruskal-Wallis rank sum test | 0.004 |
| Mean (SD) | 0.88 (0.10) | 0.95 (0.04) | 0.92 (0.08) |  |  |
| Median (Q1, Q3) | 0.91 (0.83, 0.95) | 0.96 (0.93, 0.97) | 0.95 (0.90, 0.97) |  |  |
| Min - Max | 0.65 - 0.97 | 0.82 - 0.98 | 0.65 - 0.98 |  |  |
| **G-mean** |  |  |  | Kruskal-Wallis rank sum test | 0.264 |
| Mean (SD) | 0.65 (0.19) | 0.72 (0.15) | 0.69 (0.17) |  |  |
| Median (Q1, Q3) | 0.77 (0.55, 0.78) | 0.73 (0.67, 0.83) | 0.73 (0.59, 0.79) |  |  |
| Min - Max | 0.29 - 0.89 | 0.30 - 0.93 | 0.29 - 0.93 |  |  |
| **F-value** |  |  |  | Kruskal-Wallis rank sum test | 0.020 |
| Mean (SD) | 0.94 (0.02) | 0.96 (0.01) | 0.95 (0.02) |  |  |
| Median (Q1, Q3) | 0.95 (0.94, 0.95) | 0.96 (0.95, 0.97) | 0.95 (0.94, 0.96) |  |  |
| Min - Max | 0.91 - 0.98 | 0.93 - 0.98 | 0.91 - 0.98 |  |  |
| **Sensitivity** |  |  |  | Kruskal-Wallis rank sum test | 0.012 |
| Mean (SD) | 0.95 (0.02) | 0.97 (0.01) | 0.96 (0.02) |  |  |
| Median (Q1, Q3) | 0.95 (0.93, 0.96) | 0.98 (0.95, 0.98) | 0.96 (0.95, 0.98) |  |  |
| Min - Max | 0.92 - 0.98 | 0.95 - 0.99 | 0.92 - 0.99 |  |  |
| **Precision** |  |  |  | Linear Model ANOVA | 0.207 |
| Mean (SD) | 0.93 (0.03) | 0.94 (0.02) | 0.94 (0.03) |  |  |
| Median (Q1, Q3) | 0.95 (0.91, 0.95) | 0.94 (0.93, 0.96) | 0.94 (0.92, 0.95) |  |  |
| Min - Max | 0.89 - 0.98 | 0.89 - 0.99 | 0.89 - 0.99 |  |  |
| **Specificity** |  |  |  | Kruskal-Wallis rank sum test | 0.378 |
| Mean (SD) | 0.48 (0.23) | 0.56 (0.19) | 0.53 (0.21) |  |  |
| Median (Q1, Q3) | 0.64 (0.32, 0.64) | 0.55 (0.45, 0.73) | 0.55 (0.36, 0.64) |  |  |
| Min - Max | 0.09 - 0.82 | 0.09 - 0.91 | 0.09 - 0.91 |  |  |
| **Accuracy** |  |  |  | Kruskal-Wallis rank sum test | 0.028 |
| Mean (SD) | 0.90 (0.04) | 0.92 (0.03) | 0.91 (0.03) |  |  |
| Median (Q1, Q3) | 0.91 (0.88, 0.92) | 0.93 (0.91, 0.94) | 0.92 (0.89, 0.94) |  |  |
| Min - Max | 0.83 - 0.96 | 0.87 - 0.96 | 0.83 - 0.96 |  |  |

**Table S9 The 7 most accurate rules and intergroup comparisons of *cyp1a2*, *cyp2c19* and *il6***

| **len** | **freq** | **err** | | **condition** | | | | | **pred** |
| --- | --- | --- | --- | --- | --- | --- | --- | --- | --- |
| 3 | 0.046 | 0.000 | | cyp1a2>12201.5 & cyp2c19<=103.5 & il6<=48.5 | | | | | HCC |
| 4 | 0.024 | 0.000 | | cyp1a2>2430.5 & cyp2c19>8.5 & il6>7.5 & il6<=16 | | | | | Normal |
| 2 | 0.07 | 0.043 | | cyp1a2<=6985 & il6>52.5 | | | | | HCC |
| 1 | 0.763 | 0.048 | | cyp2c19<=164 | | | | | HCC |
| 3 | 0.027 | 0.111 | | cyp1a2>5124 & cyp2c19>26 & il6>27.5 | | | | | Normal |
| 1 | 0.036 | 0.167 | | cyp1a2>10594 | | | | | Normal |
| 1 | 0.033 | 0.000 | | Else | | | | | HCC |
|  | | | | | | | | | |
| **Gene** | | | **HCC (N=374)** | | **Normal (N=50)** | **Total (N=424)** | **Group test** | **P value** | |
| **cyp1a2** | | |  | |  |  | Kruskal-Wallis rank sum test | < 0.001 | |
| Mean (SD) | | | 3723.58 (13095.66) | | 24677.46 (17299.11) | 6194.56 (15220.70) |  |  | |
| Median (Q1, Q3) | | | 89.50 (8.00, 1288.75) | | 22216.50 (10338.50, 31273.75) | 174.00 (10.00, 3349.25) |  |  | |
| Min - Max | | | 0.00 - 136925.00 | | 2597.00 - 70947.00 | 0.00 - 136925.00 |  |  | |
| **cyp2c19** | | |  | |  |  | Kruskal-Wallis rank sum test | < 0.001 | |
| Mean (SD) | | | 55.33 (270.46) | | 467.64 (657.11) | 103.95 (363.65) |  |  | |
| Median (Q1, Q3) | | | 13.00 (5.00, 26.00) | | 211.00 (33.00, 704.25) | 15.00 (6.00, 33.00) |  |  | |
| Min - Max | | | 0.00 - 4288.00 | | 6.00 - 3214.00 | 0.00 - 4288.00 |  |  | |
| **il6** | | |  | |  |  | Kruskal-Wallis rank sum test | < 0.001 | |
| Mean (SD) | | | 33.66 (93.22) | | 96.48 (241.93) | 41.07 (121.88) |  |  | |
| Median (Q1, Q3) | | | 8.00 (3.00, 22.75) | | 26.50 (9.25, 62.50) | 9.00 (3.00, 25.00) |  |  | |
| Min - Max | | | 0.00 - 789.00 | | 0.00 - 1559.00 | 0.00 - 1559.00 |  |  | |

The selected rules consist of conditions and predictions (denoted as “pred”), and the following rule metrics: length (denoted as “len”), frequency (denoted as “freq”), and error (denoted as “err”).

**Table S10 Topological parameters of the 15 genes located in the *OAMs***

| ***OAMs*** |  | **Genes** | **Betweenness** | **Bridging** | **Centroid** | **Closeness** | **Degree** | **Eccentricity** | **EigenVector** | **Radiality** | **Stress** |
| --- | --- | --- | --- | --- | --- | --- | --- | --- | --- | --- | --- |
| ***AM^O^_CHB11-HCC6_*** | **CHB11** | cyp2c9 | 0.3333333 | 0.0454545 | 0 | 0.1428571 | 7 | 1 | 0.3634563 | 2 | 2 |
|  |  | cyp2c19 | 0.3333333 | 0.0454545 | 0 | 0.1428571 | 7 | 1 | 0.3634563 | 2 | 2 |
|  |  | cyp1a2 | 0.3333333 | 0.0454545 | 0 | 0.1428571 | 7 | 1 | 0.3634563 | 2 | 2 |
|  |  | cyp3a4 | 0.3333333 | 0.0454545 | 0 | 0.1428571 | 7 | 1 | 0.3634563 | 2 | 2 |
|  |  | cyp2a6 | 0.3333333 | 0.0454545 | 0 | 0.1428571 | 7 | 1 | 0.3634563 | 2 | 2 |
|  |  | cyp2e1 | 0.3333333 | 0.0454545 | 0 | 0.1428571 | 7 | 1 | 0.3634563 | 2 | 2 |
|  |  | cyp2d6 | 0 | 0 | -1 | 0.125 | 6 | 0.5 | 0.3220226 | 1.8571429 | 0 |
|  |  | ercc8 | 0 | 0 | -1 | 0.125 | 6 | 0.5 | 0.3220226 | 1.8571429 | 0 |
|  |  |  |  |  |  |  |  |  |  |  |  |
|  | **HCC6** | cyp3a | 0.2857143 | 0.0344828 | 0 | 0.125 | 8 | 1 | 0.3407495 | 2 | 2 |
|  |  | cyp2a6 | 0.2857143 | 0.0344828 | 0 | 0.125 | 8 | 1 | 0.3407495 | 2 | 2 |
|  |  | cyp2b6 | 0.2857143 | 0.0344828 | 0 | 0.125 | 8 | 1 | 0.3407495 | 2 | 2 |
|  |  | cyp2c8 | 0.2857143 | 0.0344828 | 0 | 0.125 | 8 | 1 | 0.3407495 | 2 | 2 |
|  |  | cyp2e1 | 0.2857143 | 0.0344828 | 0 | 0.125 | 8 | 1 | 0.3407495 | 2 | 2 |
|  |  | cyp2c9 | 0.2857143 | 0.0344828 | 0 | 0.125 | 8 | 1 | 0.3407495 | 2 | 2 |
|  |  | cyp2c19 | 0.2857143 | 0.0344828 | 0 | 0.125 | 8 | 1 | 0.3407495 | 2 | 2 |
|  |  | cyp1a2 | 0 | 0 | -1 | 0.1111111 | 7 | 0.5 | 0.3059644 | 1.875 | 0 |
|  |  | cyp2d6 | 0 | 0 | -1 | 0.1111111 | 7 | 0.5 | 0.3059644 | 1.875 | 0 |
|  |  |  |  |  |  |  |  |  |  |  |  |
| ***AM^O^_CHB7-HCC20,_ AM^O^_C2-HCC20_*** | **HCC20** | akt1 | 36.4 | 1.9569892 | 3 | 0.1111111 | 9 | 1 | 0.4245072 | 2 | 38 |
|  |  | abcb1 | 0.4 | 0.0659341 | -3 | 0.0833333 | 6 | 0.5 | 0.3766645 | 1.6666667 | 2 |
|  |  | mdm4 | 0.4 | 0.0659341 | -3 | 0.0833333 | 6 | 0.5 | 0.3766645 | 1.6666667 | 2 |
|  |  | abcg2 | 0.4 | 0.0659341 | -3 | 0.0833333 | 6 | 0.5 | 0.3766645 | 1.6666667 | 2 |
|  |  | abcb6 | 0.4 | 0.0659341 | -3 | 0.0833333 | 6 | 0.5 | 0.3766645 | 1.6666667 | 2 |
|  |  | pi3 | 0 | 0 | -4 | 0.0769231 | 5 | 0.5 | 0.3288219 | 1.5555556 | 0 |
|  |  | saa@ | 0 | 0 | -4 | 0.0769231 | 5 | 0.5 | 0.3288219 | 1.5555556 | 0 |
|  |  | vegfa | 0 | 0 | -6 | 0.0666667 | 3 | 0.5 | 0.1096073 | 1.3333333 | 0 |
|  |  | il8 | 0 | 0 | -6 | 0.0666667 | 3 | 0.5 | 0.1096073 | 1.3333333 | 0 |
|  |  | il6 | 0 | 0 | -6 | 0.0666667 | 3 | 0.5 | 0.1096073 | 1.3333333 | 0 |
|  |  |  |  |  |  |  |  |  |  |  |  |
| ***AM^O^_CHB36-HCC3_*** | **CHB36** | decr1 | 1 | 0.25 | 0 | 0.333333 | 3 | 1 | 0.557345 | 2 | 2 |
|  |  | ros1 | 1 | 0.25 | 0 | 0.333333 | 3 | 1 | 0.557345 | 2 | 2 |
|  |  | apoe | 0 | 0 | -1 | 0.25 | 2 | 0.5 | 0.435162 | 1.666667 | 0 |
|  |  | syk | 0 | 0 | -1 | 0.25 | 2 | 0.5 | 0.435162 | 1.666667 | 0 |
|  |  |  |  |  |  |  |  |  |  |  |  |
|  | **HCC3** | nox1 | 0.2 | 0.017857 | 0 | 0.090909 | 11 | 1 | 0.292344 | 2 | 2 |
|  |  | lyn | 0.2 | 0.017857 | 0 | 0.090909 | 11 | 1 | 0.292344 | 2 | 2 |
|  |  | crp | 0.2 | 0.017857 | 0 | 0.090909 | 11 | 1 | 0.292344 | 2 | 2 |
|  |  | pkc | 0.2 | 0.017857 | 0 | 0.090909 | 11 | 1 | 0.292344 | 2 | 2 |
|  |  | syk | 0.2 | 0.017857 | 0 | 0.090909 | 11 | 1 | 0.292344 | 2 | 2 |
|  |  | rac1 | 0.2 | 0.017857 | 0 | 0.090909 | 11 | 1 | 0.292344 | 2 | 2 |
|  |  | ptk2 | 0.2 | 0.017857 | 0 | 0.090909 | 11 | 1 | 0.292344 | 2 | 2 |
|  |  | gp6 | 0.2 | 0.017857 | 0 | 0.090909 | 11 | 1 | 0.292344 | 2 | 2 |
|  |  | ros1 | 0.2 | 0.017857 | 0 | 0.090909 | 11 | 1 | 0.292344 | 2 | 2 |
|  |  | tgfb1i1 | 0.2 | 0.017857 | 0 | 0.090909 | 11 | 1 | 0.292344 | 2 | 2 |
|  |  | btk | 0 | 0 | -1 | 0.083333 | 10 | 0.5 | 0.269583 | 1.909091 | 0 |
|  |  | pik3ca | 0 | 0 | -1 | 0.083333 | 10 | 0.5 | 0.269583 | 1.909091 | 0 |
|  |  |  |  |  |  |  |  |  |  |  |  |
| ***AM^O^_CHB14-HCC21_*** | **CHB14** | znf296 | 0.4 | 0.0625 | 0 | 0.166667 | 6 | 1 | 0.39173 | 2 | 2 |
|  |  | mgmt | 0.4 | 0.0625 | 0 | 0.166667 | 6 | 1 | 0.39173 | 2 | 2 |
|  |  | socs1 | 0.4 | 0.0625 | 0 | 0.166667 | 6 | 1 | 0.39173 | 2 | 2 |
|  |  | rfx1 | 0.4 | 0.0625 | 0 | 0.166667 | 6 | 1 | 0.39173 | 2 | 2 |
|  |  | pycard | 0.4 | 0.0625 | 0 | 0.166667 | 6 | 1 | 0.39173 | 2 | 2 |
|  |  | rassf1 | 0 | 0 | -1 | 0.142857 | 5 | 0.5 | 0.34113 | 1.833333 | 0 |
|  |  | emp3 | 0 | 0 | -1 | 0.142857 | 5 | 0.5 | 0.34113 | 1.833333 | 0 |
|  |  |  |  |  |  |  |  |  |  |  |  |
|  | **HCC21** | rassf1 | 0.5 | 0.067568 | 0 | 0.2 | 6 | 1 | 0.428445 | 2 | 2 |
|  |  | mgmt | 0.5 | 0.067568 | 0 | 0.2 | 6 | 1 | 0.428445 | 2 | 2 |
|  |  | socs3 | 0.5 | 0.096774 | 0 | 0.2 | 5 | 1 | 0.428445 | 2 | 2 |
|  |  | pgr | 0.5 | 0.096774 | 0 | 0.2 | 5 | 1 | 0.428445 | 2 | 2 |
|  |  | fhit | 0 | 0 | -1 | 0.166667 | 4 | 0.5 | 0.364513 | 1.8 | 0 |
|  |  | ptgs2 | 0 | 0 | -1 | 0.166667 | 4 | 0.5 | 0.364513 | 1.8 | 0 |
|  |  |  |  |  |  |  |  |  |  |  |  |
| ***AM^O^_CHB9-HCC12_*** | **CHB9** | xiap | 0.285714 | 0.034483 | 0 | 0.125 | 8 | 1 | 0.34075 | 2 | 2 |
|  |  | ddit3 | 0.285714 | 0.034483 | 0 | 0.125 | 8 | 1 | 0.34075 | 2 | 2 |
|  |  | hspa5 | 0.285714 | 0.034483 | 0 | 0.125 | 8 | 1 | 0.34075 | 2 | 2 |
|  |  | tnfsf10 | 0.285714 | 0.034483 | 0 | 0.125 | 8 | 1 | 0.34075 | 2 | 2 |
|  |  | diablo | 0.285714 | 0.034483 | 0 | 0.125 | 8 | 1 | 0.34075 | 2 | 2 |
|  |  | col9a1 | 0.285714 | 0.034483 | 0 | 0.125 | 8 | 1 | 0.34075 | 2 | 2 |
|  |  | bcl2l1 | 0.285714 | 0.034483 | 0 | 0.125 | 8 | 1 | 0.34075 | 2 | 2 |
|  |  | tnfrsf10b | 0 | 0 | -1 | 0.111111 | 7 | 0.5 | 0.305964 | 1.875 | 0 |
|  |  | bax | 0 | 0 | -1 | 0.111111 | 7 | 0.5 | 0.305964 | 1.875 | 0 |
|  |  |  |  |  |  |  |  |  |  |  |  |
|  | **HCC12** | ebp | 0.333333 | 0.045455 | 0 | 0.142857 | 7 | 1 | 0.363456 | 2 | 2 |
|  |  | ddit3 | 0.333333 | 0.045455 | 0 | 0.142857 | 7 | 1 | 0.363456 | 2 | 2 |
|  |  | atf6 | 0.333333 | 0.045455 | 0 | 0.142857 | 7 | 1 | 0.363456 | 2 | 2 |
|  |  | ern1 | 0.333333 | 0.045455 | 0 | 0.142857 | 7 | 1 | 0.363456 | 2 | 2 |
|  |  | mapk8 | 0.333333 | 0.045455 | 0 | 0.142857 | 7 | 1 | 0.363456 | 2 | 2 |
|  |  | hspa5 | 0.333333 | 0.045455 | 0 | 0.142857 | 7 | 1 | 0.363456 | 2 | 2 |
|  |  | bcl2 | 0 | 0 | -1 | 0.125 | 6 | 0.5 | 0.322023 | 1.857143 | 0 |
|  |  | bax | 0 | 0 | -1 | 0.125 | 6 | 0.5 | 0.322023 | 1.857143 | 0 |
|  |  |  |  |  |  |  |  |  |  |  |  |
| ***AM^O^_CHB7-HCC3_*** | **CHB7** | mapk3 | 0.285714 | 0.028021 | 0 | 0.125 | 9 | 1 | 0.34075 | 2 | 2 |
|  |  | pik3ca | 0.285714 | 0.028021 | 0 | 0.125 | 9 | 1 | 0.34075 | 2 | 2 |
|  |  | mmp2 | 0.285714 | 0.035433 | 0 | 0.125 | 8 | 1 | 0.34075 | 2 | 2 |
|  |  | ptk2 | 0.285714 | 0.035433 | 0 | 0.125 | 8 | 1 | 0.34075 | 2 | 2 |
|  |  | il8 | 0.285714 | 0.035433 | 0 | 0.125 | 8 | 1 | 0.34075 | 2 | 2 |
|  |  | akt1 | 0.285714 | 0.035433 | 0 | 0.125 | 8 | 1 | 0.34075 | 2 | 2 |
|  |  | ccl2 | 0.285714 | 0.035433 | 0 | 0.125 | 8 | 1 | 0.34075 | 2 | 2 |
|  |  | lyn | 0 | 0 | -1 | 0.111111 | 7 | 0.5 | 0.305964 | 1.875 | 0 |
|  |  | vegfa | 0 | 0 | -1 | 0.111111 | 7 | 0.5 | 0.305964 | 1.875 | 0 |
|  |  |  |  |  |  |  |  |  |  |  |  |
| ***AM^O^_CHB23-C11-HCC38_*** | **CHB23** | sirt1 | 88 | 7.652174 | 1 | 0.055556 | 6 | 0.5 | 0.125832 | 3.5 | 120 |
|  |  | hdac2 | 70 | 10 | -1 | 0.052632 | 6 | 0.333333 | 0.420045 | 3.416667 | 90 |
|  |  | hdac4 | 0 | 0 | -7 | 0.038462 | 5 | 0.25 | 0.399253 | 2.833333 | 0 |
|  |  | krt16 | 0 | 0 | -7 | 0.038462 | 5 | 0.25 | 0.399253 | 2.833333 | 0 |
|  |  | krt18 | 0 | 0 | -7 | 0.038462 | 5 | 0.25 | 0.399253 | 2.833333 | 0 |
|  |  | trps1 | 0 | 0 | -7 | 0.038462 | 5 | 0.25 | 0.399253 | 2.833333 | 0 |
|  |  | hdac9 | 0 | 0 | -7 | 0.038462 | 5 | 0.25 | 0.399253 | 2.833333 | 0 |
|  |  | ppargc1a | 6 | 1.5 | -7 | 0.04 | 4 | 0.333333 | 0.051188 | 2.916667 | 18 |
|  |  | rnr1 | 6 | 1.5 | -7 | 0.04 | 4 | 0.333333 | 0.051188 | 2.916667 | 18 |
|  |  | pparg | 6 | 1.5 | -7 | 0.04 | 4 | 0.333333 | 0.051188 | 2.916667 | 18 |
|  |  | tfam | 0 | 0 | -9 | 0.029412 | 3 | 0.25 | 0.030396 | 2.166667 | 0 |
|  |  | aprt | 0 | 0 | -10 | 0.035714 | 2 | 0.333333 | 0.031054 | 2.666667 | 0 |
|  |  | prkaa1 | 0 | 0 | -10 | 0.035714 | 2 | 0.333333 | 0.031054 | 2.666667 | 0 |
|  |  |  |  |  |  |  |  |  |  |  |  |
|  | **C11** | hdac4 | 0.5 | 0.090909 | 0 | 0.2 | 5 | 1 | 0.428445 | 2 | 2 |
|  |  | hdac9 | 0.5 | 0.090909 | 0 | 0.2 | 5 | 1 | 0.428445 | 2 | 2 |
|  |  | krt18 | 0.5 | 0.090909 | 0 | 0.2 | 5 | 1 | 0.428445 | 2 | 2 |
|  |  | trps1 | 0.5 | 0.090909 | 0 | 0.2 | 5 | 1 | 0.428445 | 2 | 2 |
|  |  | hdac2 | 0 | 0 | -1 | 0.166667 | 4 | 0.5 | 0.364513 | 1.8 | 0 |
|  |  | krt16 | 0 | 0 | -1 | 0.166667 | 4 | 0.5 | 0.364513 | 1.8 | 0 |
|  |  |  |  |  |  |  |  |  |  |  |  |
|  | **HCC38** | hdac10 | 24 | 3.2 | 2 | 0.1 | 5 | 0.333333 | 0.462834 | 3.571429 | 24 |
|  |  | hdac3 | 0 | 0 | -3 | 0.076923 | 4 | 0.25 | 0.438692 | 3.142857 | 0 |
|  |  | hdac2 | 0 | 0 | -3 | 0.076923 | 4 | 0.25 | 0.438692 | 3.142857 | 0 |
|  |  | hdac9 | 0 | 0 | -3 | 0.076923 | 4 | 0.25 | 0.438692 | 3.142857 | 0 |
|  |  | hdac6 | 0 | 0 | -3 | 0.076923 | 4 | 0.25 | 0.438692 | 3.142857 | 0 |
|  |  | mmp2 | 20 | 14.28571 | -2 | 0.083333 | 2 | 0.5 | 0.122041 | 3.285714 | 20 |
|  |  | timp2 | 12 | 4 | -4 | 0.0625 | 2 | 0.333333 | 0.032045 | 2.714286 | 12 |
|  |  | cdk5rap3 | 0 | 0 | -6 | 0.045455 | 1 | 0.25 | 0.007903 | 1.857143 | 0 |

**Table S11 The top three compounds that affected the three genes (*cyp1a2, cyp2c19* and *il6*)**

| **Compounds** | **OB** | **Drug-likeness** | **Corresponding herbs** | **The relationships between compounds and genes** |
| --- | --- | --- | --- | --- |
| **Glycyrol** | 90.77578 | 0.66819 | Licorice | Glycyrol inhibits the reaction [Lipopolysaccharides results in increased expression of ***il6*** mRNA] (1) |
| **Inermin** | 65.83093 | 0.53754 | Sophorae Flavescentis Radix, Sophorae Tonkinensis Radix Et Rhizome | Inermin results in decreased activity of ***cyp2c19*** protein (2) |
| **Bilobalide** | 84.41507 | 0.35747 | Ginkgo Folium | Bilobalide results in increased expression of and results in increased activity of ***cyp1a2*** protein. Bilobalide results in increased expression of ***cyp1a2*** mRNA (3) |

**References**

1. Satarug S, Tassaneeyakul W, Na-Bangchang K, Cashman JR, Moore MR. Genetic and environmental influences on therapeutic and toxicity outcomes: studies with CYP2A6. Curr Clin Pharmacol. 2006; 1: 291-309.
2. Li Y, Ning J, Wang Y, Wang C, Sun C, Huo X, Yu Z, Feng L, Zhang B, Tian X, Ma X. Drug interaction study of flavonoids toward CYP3A4 and their quantitative structure activity relationship (QSAR) analysis for predicting potential effects. Toxicol Lett. 2018; 294:27-36.
3. Deng Y, Bi HC, Zhao LZ, He F, Liu YQ, Yu JJ, Ou ZM, Ding L, Chen X, Huang ZY, Huang M, Zhou SF. Induction of cytochrome P450s by terpene trilactones and flavonoids of the Ginkgo biloba extract EGb 761 in rats. Xenobiotica. 2008; 38:465-81.
